# Reinforcement learning model for optimizing dexmedetomidine dosing to prevent delirium in critically ill patients

**DOI:** 10.1038/s41746-024-01335-x

**Published:** 2024-11-18

**Authors:** Hong Yeul Lee, Soomin Chung, Dongwoo Hyeon, Hyun-Lim Yang, Hyung-Chul Lee, Ho Geol Ryu, Hyeonhoon Lee

**Affiliations:** 1 Department of Critical Care Medicine, Seoul National University Hospital, Seoul, Republic of Korea; 2 Interdisciplinary Program in Bioengineering, Seoul National University, Seoul, Republic of Korea; 3 Biomedical Research Institute, Seoul National University Hospital, Seoul, Republic of Korea; 4 Office of Hospital Information, Seoul National University Hospital, Seoul, Republic of Korea; 5 Department of Medical Device Development Support, Innovative Medical Technology Research Institute, Seoul National University Hospital, Seoul, Republic of Korea; 6 Department of Medicine, Seoul National University College of Medicine, Seoul, Republic of Korea; 7 Department of Anesthesiology and Pain Medicine, Seoul National University College of Medicine, Seoul National University Hospital, Seoul, Republic of Korea; 8 Department of Data Science Research, Innovative Medical Technology Research Institute, Seoul National University Hospital, Seoul, Republic of Korea

**Keywords:** Health care, Medical research

## Abstract

Delirium can result in undesirable outcomes including increased length of stays and mortality in patients admitted to the intensive care unit (ICU). Dexmedetomidine has emerged for delirium prevention in these patients; however, optimal dosing is challenging. A reinforcement learning-based Artificial Intelligence model for Delirium prevention (AID) is proposed to optimize dexmedetomidine dosing. The model was developed and internally validated using 2416 patients (2531 ICU admissions) and externally validated on 270 patients (274 ICU admissions). The estimated performance return of the AID policy was higher than that of the clinicians’ policy in both derivation (0.390 95% confidence interval [CI] 0.361 to 0.420 vs. −0.051 95% CI −0.077 to −0.025) and external validation (0.186 95% CI 0.139 to 0.236 vs. −0.436 95% CI −0.474 to −0.402) cohorts. Our finding indicates that AID might support clinicians’ decision-making regarding dexmedetomidine dosing to prevent delirium in ICU patients, but further off-policy evaluation is required.

## Introduction

Delirium is a complex neuropsychiatric syndrome characterized by acute fluctuations in attention, awareness, and cognition^1^, with a prevalence of 20%–80% among critically ill patients^2^. It is associated with poor clinical outcomes, including increased in-hospital mortality, long-term cognitive decline, and a longer duration of mechanical ventilation and intensive care unit (ICU) stay^3,4^. Therefore, preventing delirium is crucial for improving patient prognosis.

Recently, supervised learning-based machine learning models have been developed to predict the onset of delirium using routinely collected electronic medical records (EMRs)^5–7^. Although these models can effectively forecast the likelihood of delirium over time, they primarily serve as diagnostic or alert tools. Their main strength lies in leveraging EMR data; however their scope remains limited to outcome prediction offering solely an early warning, and they do not provide specific guidance on interventions. To bridge this gap, an “actionable” model has been proposed. This model can predict future patient outcomes or events resulting from different treatment options, thereby advising clinicians on treatment options that yield the best predictive outcome^8^.

Dexmedetomidine, a high-affinity alpha-2 adrenergic agonist, holds promise in managing critically ill patients, particularly for delirium prevention^9,10^. It provides sedation with less respiratory depression, making it a favorable choice over traditional sedatives such as benzodiazepines and propofol^11^. A recent trial has presented its potential benefits in reducing the incidence of delirium compared to usual care sedatives in mechanically ventilated ICU patients^12^. However, dexmedetomidine requires clinicians to monitor and adjust dosages carefully due to potential adverse events such as bradycardia and hypotension^11^. Nonetheless, there is a lack of clear guidelines for the optimal dosage of dexmedetomidine, posing challenges in clinical practices^13^.

Traditional dosing strategies largely rely on empirical knowledge due to the absence of a universally accepted consensus or specific guidelines on dexmedetomidine dosing. Previous studies generally recommend an initial dosing rate of 0.2–0.4 mcg/kg/h and suggest titration adjustments of 0.1–0.2 mcg/kg/h, without providing guidance on specific dosages responding to different patient conditions^9,14–16^. Therefore, these traditional dosing strategies often fail to adequately address the dynamic nature of patient responses in intricate ICU environments, necessitating more adaptive approaches.

Reinforcement learning, which is a branch of machine learning, offers a potential solution to this challenge^17^. Reinforcement learning aims to identify the best decision-making policy by considering future cumulative rewards. Previous studies based on reinforcement learning algorithms have proposed optimal drug dosing policies aimed at preventing mortality or hypotension in ICU settings^18–21^. Similarly, a reinforcement learning model can provide sequential dosing recommendations to prevent the development of delirium throughout the ICU stay. In a recent study, the use of a reinforcement learning algorithm for delirium prevention has been explored by suggesting whether to increase, decrease, or maintain the dosage of propofol, midazolam, and fentanyl^22^. However, the model has limitations, such as not directly adjusting the medication dosages, and it focuses on other traditional medications that may be less effective than dexmedetomidine in preventing delirium^23^.

The primary objective of this study is to develop and validate a reinforcement learning-based Artificial Intelligence model for Delirium prevention (AID) by optimizing dexmedetomidine dosing in critically ill patients to prevent the development of delirium during their ICU stays. We hypothesize that compared to the clinicians’ policy, the policy suggested by AID would yield a higher estimated performance return defined by the onset of delirium, resulting in a reduced incidence of delirium.

## Results

### Dataset construction

**Fig. 1:**
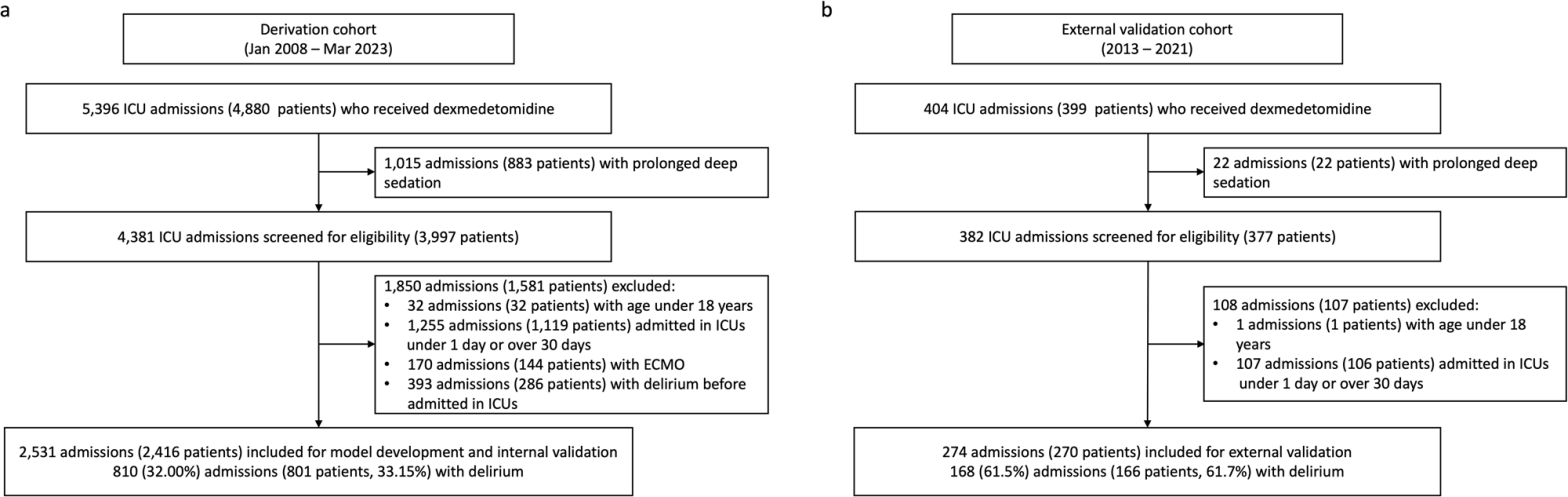
Flow chart of dataset construction. **a** Derivation cohort. **b** External validation cohort.

Among the 3997 patients with 4381 ICU admissions from the derivation cohort, 2416 patients with 2531 ICU admissions (42,863 6-h interval time points) were included in the model development and internal validation (Fig. 1). In the external validation cohort, 270 patients with 274 ICU admissions (2009 6-h interval time points) were included for the external validation after applying the exclusion criteria. The characteristics of the analyzed admissions are listed in Table 1.

**Table 1:** Patient characteristics at ICU admission levels

	Derivation cohort (*n* = 2531)			External validation cohort (*n* = 274)		
	With delirium (*n* = 810)	Without delirium (*n* = 1721)	*P*-value	With delirium (*n* = 168)	Without delirium (*n* = 106)	*P*-value
Age (year), median (IQR)	67.0 (56.2–76.0)	67.0 (56.0–75.0)	0.103	70.0 (60.0–80.0)	72.5 (61.2–80.0)	0.644
Sex, *n* (%)			0.415			0.098
Female	286 (35.3)	638 (37.1)		52 (31.0)	44 (41.5)	
Male	524 (64.7)	1083 (62.9)		116 (69.0)	62 (58.5)	
ICU type, *n* (%)			<0.001	NA	NA	
Medical	251 (31.0)	730 (42.4)				
Surgical	559 (69.0)	991 (57.6)				
Height (cm), median (IQR)	165.0 (156.3–170.0)	163.0 (156.0–170.0)	0.484	170.0 (165.0–176.2)	170.0 (165.0–175.0)	0.070
Weight (kg), median (IQR)	61.5 (53.6–71.2)	60.4 (52.3–69.3)	0.013	75.0 (65.0–86.2)	75.0 (65.0–90.0)	0.771
ICU length of stay (day), median (IQR)	7 (4–13)	5 (2–9)	<0.001	7 (4–12)	3 (1–7)	<0.001
SOFA score on the first day of dexmedetomidine usage	7 (5–10)	8 (5–10)	0.013	NA	NA	
RASS score on the first day of dexmedetomidine usage, median (IQR)	−1 (-4–0)	-2 (-4–0)	<0.001	NA	NA	
Mechanical ventilation on the first day of dexmedetomidine usage, *n* (%)	794 (98.0)	1707 (99.2)	0.020	130 (77.4)	86 (81.1)	0.556
Shock, receiving vasopressors on the first day of dexmedetomidine usage, *n* (%)	303 (37.4)	775 (45.0)	<0.001	47 (28.0)	32 (30.2)	0.797
CRRT on the first day of dexmedetomidine usage, *n* (%)	136 (16.8)	356 (20.7)	0.024	9 (5.4)	4 (3.8)	0.757

Categorical variables were analyzed by proportional differences with the chi-squared test or Fisher exact test. The *t*-test and Wilcoxon rank sums test were used to compare continuous and ordinal variables, respectively.

*IQR* interquartile range, *ICU* intensive care unit, *SOFA* sequential organ failure assessment, *RASS* Richmond agitation-sedation scale, *CRRT* continuous renal replacement therapy.

### Policy and outcome differences

We conducted two different off-policy evaluations (OPEs) to compare the estimated performance return of the AID policy with that of the clinicians’ policy: a model-based approach with fitted Q-evaluation (FQE) and a model-free approach with weighted importance sampling (WIS). The FQE results showed that the estimated performance returns of the AID policy and clinicians’ policy on the aggregated internal test set were 0.390 (95% confidence interval [CI] 0.361 to 0.420) and −0.051 (95% CI −0.077 to −0.025), respectively. On the external validation cohort, the estimated performance returns of the AID policy and clinicians’ policy were 0.186 (95% CI 0.139 to 0.236) and −0.436 (95% CI −0.474 to −0.402), respectively. Notably, the 95% lower bound of the performance return of AID was higher than the 95% upper bound of the clinicians’ return in both cohorts. Using WIS, where the effective sample size was calculated as 3.33 out of 2531 admissions (0.13%), the estimated performance returns of AID and clinicians’ policies on the aggregated internal test set were –0.475 (95% CI −3.197 to 1.222) and 0.283 (95% CI 0.249 to 0.313), respectively. On the external validation cohort, where the effective sample size was calculated as 8.00 out of 274 admissions (2.92%), the estimated performance returns of the AID policy and clinicians’ policy were 0.923 (95% CI 0.005 to 2.667) and −0.251 (95% CI −0.355 to −0.139), respectively. Results from both OPEs on the individual test sets are detailed in Table 2.

**Table 2:** Estimated performance returns of clinicians’, AID, and random policy using two off-policy evaluation methods

	Clinicians’ policy			AID policy			Random policy		
	Mean	95% CI lower bound	95% CI upper bound	Mean	95% CI lower bound	95% CI upper bound	Mean	95% CI lower bound	95% CI upper bound
*Fitted-Q evaluation*									
Derivation cohort									
Fold 1	0.104	0.061	0.149	0.311	0.259	0.365	−0.230	−0.277	−0.179
Fold 2	−0.288	−0.333	−0.243	0.230	0.179	0.283	0.012	−0.026	0.052
Fold 3	−0.462	−0.511	−0.413	0.384	0.331	0.439	−0.213	−0.262	−0.163
Fold 4	0.436	0.378	0.494	0.429	0.380	0.479	0.128	0.076	0.182
Fold 5	−0.570	−0.635	−0.505	0.403	0.353	0.450	−0.484	−0.528	−0.441
Aggregated	−0.051	−0.077	−0.025	0.390	0.361	0.420	0.137	0.122	0.151
External validation cohort									
Total	−0.436	−0.474	-0.402	0.186	0.139	0.236	−0.539	−0.583	−0.499
*Weighted importance sampling*									
Derivation cohort									
Fold 1	0.277	0.203	0.347	0.910	0.001	5.343	−0.038	−0.124	−0.007
Fold 2	0.292	0.221	0.360	0.618	−2.061	3.694	0.035	−0.005	0.190
Fold 3	0.292	0.219	0.362	1.086	0.110	2.534	−0.077	−0.256	−0.015
Fold 4	0.270	0.193	0.341	0.355	−0.043	1.730	−0.009	−0.051	0.000
Fold 5	0.282	0.211	0.350	−0.902	−5.264	0.065	−0.206	−1.224	−0.002
Aggregated	0.283	0.249	0.313	−0.475	−3.197	1.222	−0.111	−0.420	−0.027
External validation cohort									
Total	−0.251	−0.355	−0.139	0.923	0.005	2.667	−0.226	−0.765	−0.024

The estimated performance returns were analyzed during 5-fold cross-validation through two off-policy evaluation methods including fitted-Q evaluation and weighted importance sampling.

*AID* artificial intelligence model for delirium prevention, *CI* confidence interval.

**Fig. 2:**
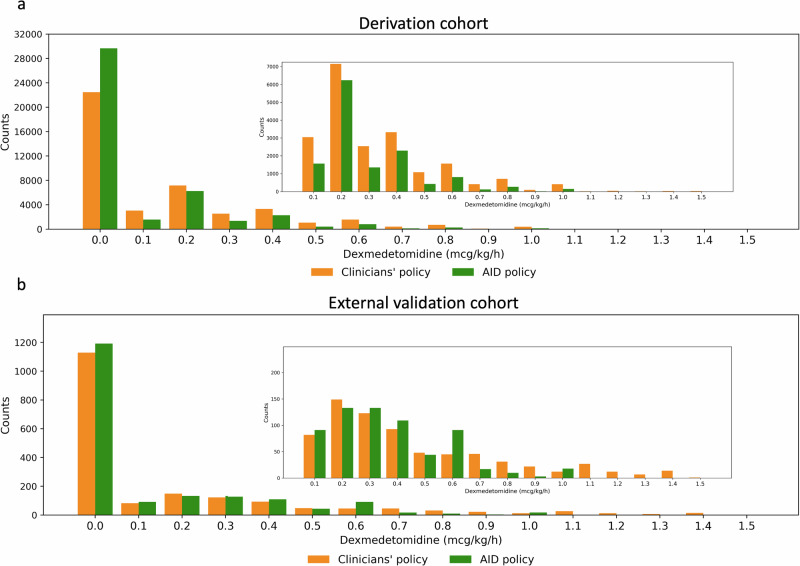
Dexmedetomidine dosing distribution of the AID policy and clinicians’ policy at all 6-h timesteps. **a** Derivation cohort. **b** External validation cohort. The outer plot shows the full range of dosages from 0.0 to 1.5 mcg/kg/h, while the inner plot focuses on the dosage range from 0.1 to 1.5 mcg/kg/h. AID artificial intelligence model for delirium prevention.

The distribution of treatment doses according to the clinicians’ and AID policies at all 6-h timesteps is presented in Fig. 2. The AID policy tends to recommend lower doses of dexmedetomidine than those administered by clinicians. Specifically, under the clinician policy, the mean dose of dexmedetomidine was 0.236 mcg/kg/h (95% CI 0.223 to 0.249) for patients who developed delirium and 0.153 mcg/kg/h (95% CI 0.145 to 0.160) for patients who did not, showing a statistically significant difference (*P* < 0.001). Under the AID policy, the mean doses were 0.117 mcg/kg/h (95% CI: 0.108 to 0.126) for patients who developed delirium and 0.090 mcg/kg/h (95% CI 0.085 to 0.094) for patients who did not, also showing a statistically significant difference *(P* = 0.001).

### Representative cases for comparison of policies

**Fig. 3:**
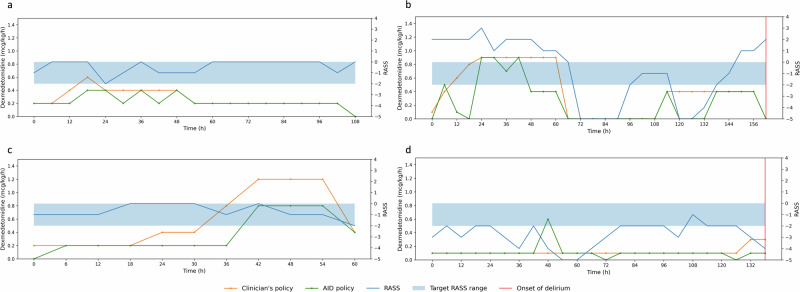
Four representative cases. **a** A case where delirium did not occur when the AID and clinicians’ policies were close. **b** A case where delirium occurred when the AID and clinicians’ policies were discrepant. **c** A case where delirium did not occur when the AID and clinicians’ policies were discrepant. **d** A case where delirium occurred when the AID and clinicians’ policies were close. The RASS ranges from −5 to 4, where higher positive scores indicate increased agitation, and lower negative scores indicate deeper sedation, with a score of 0 representing the appearance of calm and normal alertness. AID artificial intelligence model for delirium prevention, RASS Richmond agitation-sedation scale.

Figure 3 shows four representative cases to observe the development of delirium depending on the degree of dose discrepancy between the AID policy and clinicians’ policy. It also displays changes in the Richmond agitation-sedation scale (RASS) values, used to assess sedation depth and guide the titration of sedatives in critically ill patients. When clinicians administered dexmedetomidine very close to the doses suggested by AID, the delirium not occurred, with RASS values maintained within the target range (Fig. 3a). However, when clinicians administered dexmedetomidine in a manner that deviated from the AID policy, delirium occurred (Fig. 3b). On the other hand, one such case shows divergent policies but no delirium occurrence (Fig. 3c). The sedation levels were maintained within the target RASS range, yet the AID policy recommended a lower dose of dexmedetomidine compared to the clinician’s policy. This suggests that slightly lower dosages might be sufficient to prevent the development of delirium, potentially minimizing the risks of adverse effects associated with higher doses. Conversely, one such case involves similar policies where delirium occurred despite the patient receiving very low doses of dexmedetomidine during the ICU stay (Fig. 3d). Since dexmedetomidine primarily serve as a sedative, the patient already in deep sedation may not benefit from further dosage reductions. This limited capacity to lighten sedation depth potentially contributed to the failure in preventing the development of delirium.

### Feature importance analysis

**Fig. 4:**
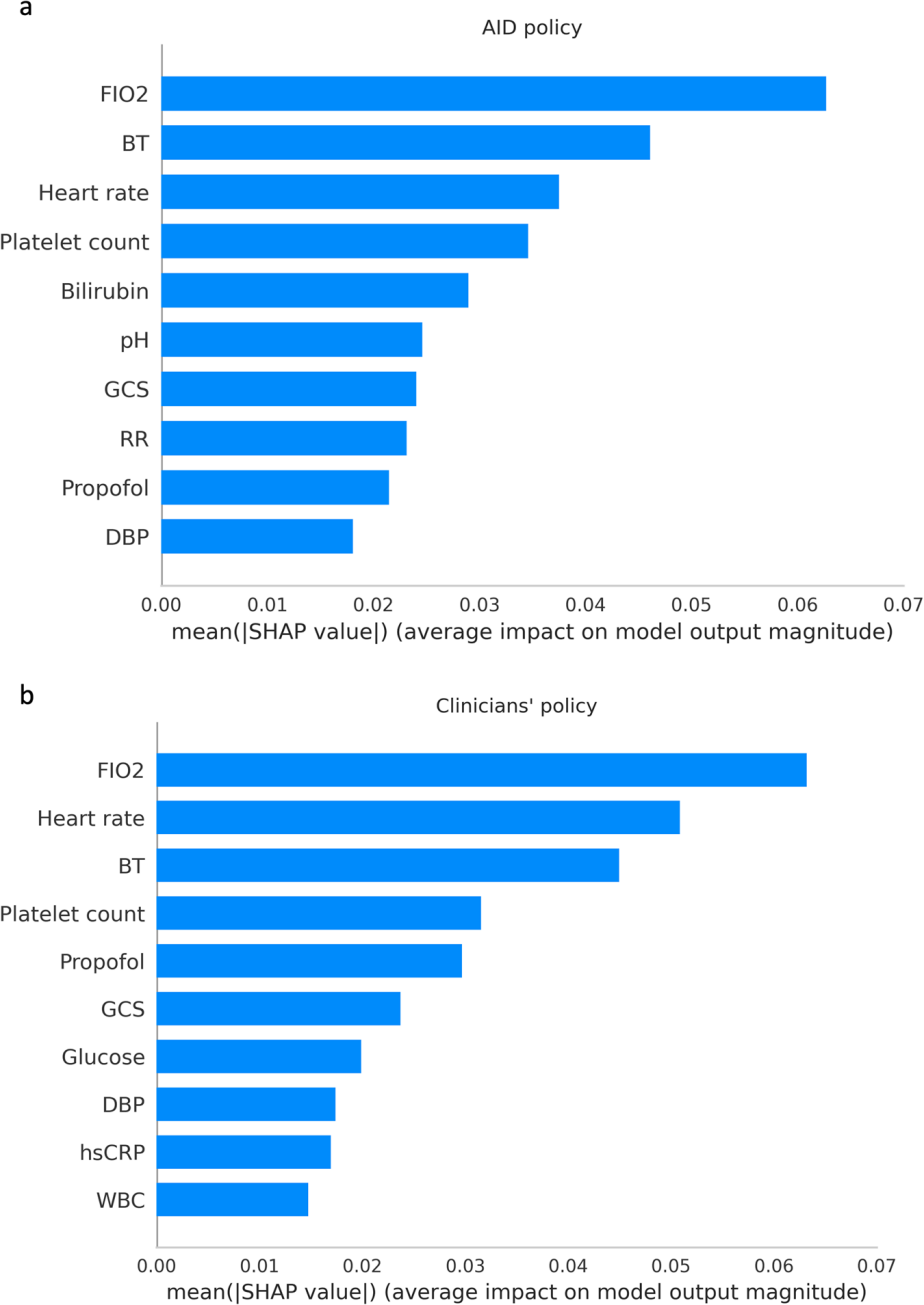
Feature importance derived from the SHAP method. **a** Feature importance of the AID policy. **b** Feature importance of the clinicians’ policy. GCS Glasgow coma scale, F_I_O2 fraction of inspired oxygen, SBP systolic blood pressure, DBP diastolic blood pressure, hsCRP high-sensitivity C-reactive protein, BT body temperature, SHAP Shapley additive explanations, AID artificial intelligence model for delirium prevention.

**Fig. 5:**
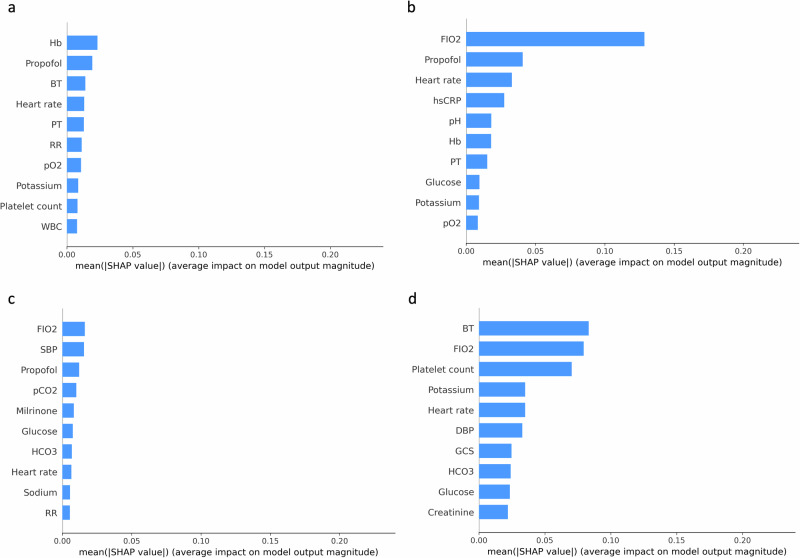
Feature importance derived from the SHAP method for subgroups stratified by policy matching and delirium occurrence. **a** Policy-matched subgroup with delirium. **b** Policy-matched subgroup without delirium. **c** Policy-unmatched subgroup with delirium. **d** Policy-unmatched subgroup without delirium. BT body temperature, F_I_O2 fraction of inspired oxygen, DBP diastolic blood pressure, GCS Glasgow coma scale, HCO_3_ bicarbonate, Hb hemoglobin, PT prothrombin time, RR respiratory rate, pO_2_ partial pressure of oxygen, WBC white blood cell count, hsCRP high-sensitivity C-reactive protein, SBP systolic blood pressure, pCO_2_ partial pressure of carbon dioxide, SHAP Shapley additive explanations.

**Fig. 6:**
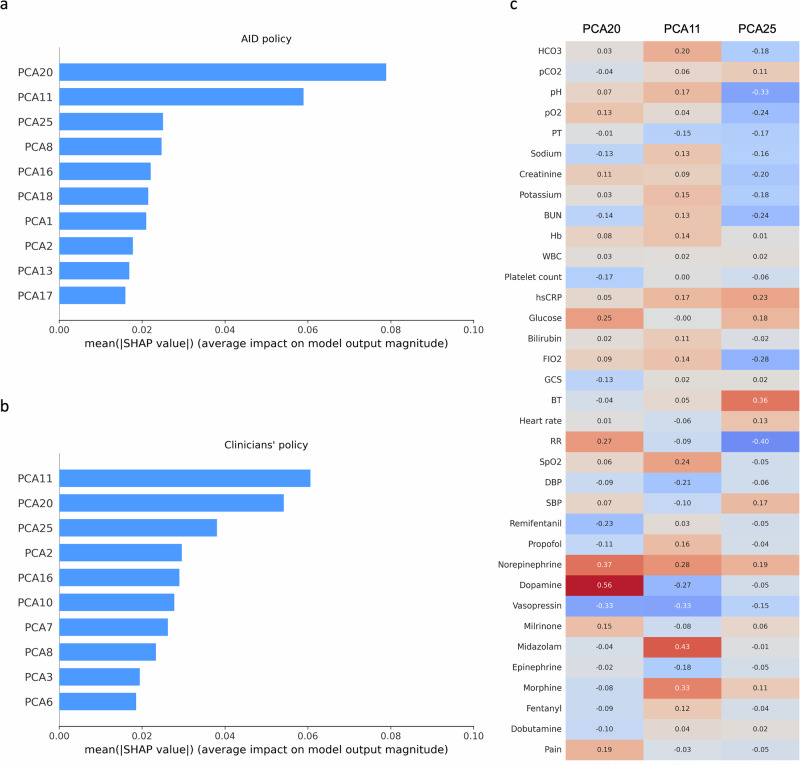
Feature importance derived from the PCA-based SHAP analysis. **a** PCA component importance of the AID policy. **b** PCA component importance of the clinicians’ policy. **c** The heatmap of the feature loadings for the top principal components in both policies. Colors represent the magnitude and direction of each feature’s contribution, with blue indicating negative loadings and red indicating positive loadings. AID artificial intelligence model for delirium prevention, HCO_3_ bicarbonate, pCO_2_ partial pressure of carbon dioxide, pO_2_ partial pressure of oxygen, PT prothrombin time, BUN blood urea nitrogen, Hb hemoglobin, WBC white blood cell count, PLT platelet count, hsCRP high-sensitivity C-reactive protein, F_I_O2 fraction of inspired oxygen, GCS glasgow coma scale, BT body temperature, RR respiratory rate, SpO_2_ oxygen saturation, DBP diastolic blood pressure, SBP systolic blood pressure, PCA principal component analysis, SHAP Shapley additive explanations.

We illustrate the degree of feature importance using the SHapley Additive exPlanations (SHAP) method for both the AID policy and clinicians’ policy, respectively. Both policies primarily considered F_I_O2, heart rate, body temperature, and platelet count for dexmedetomidine dosing (Fig. 4 and Supplementary Figs. 1–3). Beyond these four primary features, clinicians’ policies considered propofol, followed by the Glasgow coma scale (GCS). By contrast, the AID policy prioritized the bilirubin. The subgroup analyses using the SHAP method (Fig. 5 and Supplementary Fig. 4) and the pair plots (Supplementary Figs. 5 and 6) were conducted to examine differences in feature contributions and to explore the relationships among five important features where the model converged or diverged from the clinician predictions. Additionally, we employed principal component analysis (PCA) followed by the SHAP analysis to derive a theoretical framework for understanding the combination of feature importance in both policies (Fig. 6). This analysis revealed that the AID policy primarily focuses on the combination of sympathomimetic agents, followed by analgosedative agents and physiological parameters. Conversely, the clinicians’ policy, while also considering similar combinations, prioritizes analgosedative agents first, then sympathomimetic agents and physiological parameters.

## Discussion

In this study, we developed and externally validated a reinforcement learning model to optimize dexmedetomidine dosing and prevent delirium in critically ill patients. The AID policy demonstrated a superior estimated return compared with that of the clinicians’ policy, suggesting that adhering to the AID dosing recommendations could effectively prevent the development of delirium.

To the best of our knowledge, this study is the first attempt to employ a reinforcement learning algorithm at preventing delirium by managing dexmedetomidine dosing in ICU patients. By mirroring the clinicians’ management in real-world practice, we processed patient state data at 6-h intervals based on the recommendation of clinical practice guidelines to assess delirium at least once per nurse shift (e.g., every 6 to 8 h)^24,25^. As the temporal offset between the observation and dose recommendation windows narrows, there may be insufficient time to leverage pharmacological or non-pharmacological interventions for delirium^26^. Conversely, because delirium is characterized by a fluctuating course, a longer time interval (≥8 to ≥12 h) may lead to inappropriate dose recommendations. Therefore, the AID was designed to recommend a dose of dexmedetomidine every 6 h, in line with clinicians’ routine clinical practice.

The strength of our study is the generalizability of our model based on two aspects: external validation and the nature of the input data source. First, the model was validated using two independent datasets, each originating from a different hospital and country. The 95% lower bound of the FQE of the AID policy was higher than the 95% upper bound of the clinicians’ policy in both cohorts. Additionally, the 95% lower bound of the WIS of the AID policy exceeded the 95% upper bound of the clinicians’ policy in the external validation cohort, despite the small effective sample size. Second, our model was constructed using readily available data from routine EMRs. This indicates that our model is easily applicable to the prevailing hospital environment for future deployment. Furthermore, our policy model using a neural network architecture is effective in capturing the complex relationship between patient features and suggesting optimal dosing. In critical care medicine, drug dosing decisions consider multiple factors such as laboratory tests, vital signs, concurrent medications, and GCS scores. Therefore, we incorporated 35 features into the state space of the computational model.

The SHAP analysis can give us insights into how each feature contributes to the dexmedetomidine dosage decision-making. Our analysis revealed that patients receiving combined dexmedetomidine and propofol required higher dexmedetomidine doses compared to those on dexmedetomidine alone, suggesting a complex interplay between these sedatives in critical care^27^. This observation may be explained by several factors: patients needing combination therapy might have been more critically ill or had difficulties achieving desired sedation targets^28^; drug interactions could alter individual pharmacokinetics or pharmacodynamics^29^ or increased dexmedetomidine dosages may be required to counteract propofol’s hypotensive effects^30^. These findings highlight the complexity of sedation management in critically ill patients and emphasize the need for personalized, dynamic approaches to ICU sedation.

The subgroup-based SHAP analysis also reveals several differences in the model’s behavior across the four different scenarios. The model appears to overestimate certain features for the subgroups of delirium occurrence, as it heavily weighs F_I_O2 and body temperature, potentially overemphasizing respiratory and temperature control as key predictors of delirium^31,32^. For policy-divergent cases, the model successfully identifies key features like hsCRP and pH when delirium occurs, indicating recognition of systemic inflammation and metabolic disturbance^33,34^. However, in cases of policy-divergent cases without delirium, the model shifts its focus to features like F_I_O2, SBP, and pCO2, failing to capture these important inflammatory and metabolic indicators, which suggests it might not fully account for underlying pathophysiological changes. Finally, the model ranks F_I_O2 and propofol as higher important features, indicating oxygenation and the use of sedatives are primary factors in both delirium and non-delirium states^9,31,35,36^.

In our study, we applied PCA for feature extraction before conducting SHAP analysis to better understand the combinatory factors influencing our model’s decision-making process for dexmedetomidine dosing in ICU patients. The top three components were associated with sympathomimetic agents (norepinephrine and dopamine), analgosedative agents (midazolam and morphine), and physiological parameters (respiratory rate and body temperature). Notably, the PCA20 showed strong associations with norepinephrine and dopamine, key sympathomimetic agents in ICU management. These catecholamines, crucial for maintaining hemodynamic stability and organ perfusion, can indirectly affect sedation needs and delirium risk^37–39^. This finding highlights the complex interactions between sympathomimetic agents, sedatives, and patient-specific factors in ICU care^37,39^. It demonstrates the importance of personalized drug dosing strategies that balance hemodynamic support with delirium prevention, considering the impact of sympathomimetic agents and patient-specific physiological conditions.

The retrospective nature of our study imposes certain limitations on interpreting our results. In intensive care settings, where conditions are acute, it is neither feasible nor ethical to deploy unproven AI models without thorough offline validation to ensure safety and efficacy. Although reinforcement learning algorithms can potentially learn a better policy than the behavior policy when the coverage of historical data is sufficient^40^, evaluating the model in an offline setting strictly depends on OPE techniques, which come with inherent limitations. One crucial limitation is the small effective sample size, which serves as a diagnostic tool for the WIS estimator. The effective sample sizes (3 out of 2531 patients in the derivation cohort and 8 out of 274 patients in the external validation cohort) are too small to evaluate our policy with reasonable certainty, due to the substantial differences between the AID and clinicians’ policies, similar to the previous studies^17,41,42^. Therefore, further OPEs with larger datasets and a sufficient effective sample size are necessary to demonstrate that the new policy offers benefits over clinicians’ policies and to strengthen the external validation^43^.

Our model demonstrated potential through retrospective analysis and limited external validation; however, its performance is closely tied to the quality of the proxy reward distribution derived from historical clinician dosage data. In situations where clinicians are uncertain about their dosing decisions, the proxy reward distribution may be broader, leading to potential divergence in the model’s dosage recommendations. To address this issue, we employed conservative Q-learning (CQL) to mitigate the overestimation of unseen or rarely seen actions by underestimating their Q-values. Despite these efforts, extensive validation studies are necessary to establish the model’s efficacy further^44^. A prospective test-retest study is planned to evaluate real-world performance and clinician acceptance, involving a direct comparison of AI-generated recommendations with clinician decisions and an assessment of patient outcomes. If promising, a proof-of-concept feasibility trial will be considered to validate our model’s safety and effectiveness in a controlled clinical setting comprehensively.

Our external validation cohort also raised some issues due to the sample size and lack of some information. Although we used a different dataset from an independent hospital, the sample size of the external validation dataset was relatively small compared to that of the derivation dataset. Also, three of the 35 state features were unavailable. Furthermore, the lack of hospitalization times in the external validation cohort prevented excluding patients who were diagnosed with delirium prior to ICU admission, complicating the interpretation of our findings as these individuals may have different baseline risks and treatment responses.

Our study has a few additional limitations. First, the performance of reinforcement learning models is sensitive to the choice of reward function. Our study’s reward system might face a long-term credit assignment problem; therefore, incorporating an intermediate reward system based on the RASS could enable the AID policy to be more responsive and adaptive to dynamic changes in patient conditions, potentially enhancing our model’s performance^21,45^. Second, to address potential confounding, we initially identified all variables available in both datasets and selected six potential confounders based on the previous studies with clinical expertise and biological plausibility. Despite these efforts, the presence of unobservable confounders might still introduce bias into our OPE results. Therefore, future research will need to employ advanced causal inference methods, including target trial emulation, to discern causal relationships more accurately. Third, our feature importance analysis using the SHAP method, derived from a LightGBM model trained to mimic the AID and clinicians’ policies is an indirect approach. This method may not fully capture the true feature importance of the original policies and should be interpreted as an approximation rather than a direct representation of the model’s feature importance. Future work could explore novel interpretability techniques directly to reinforcement learning models for more accurate feature importance estimation.

In conclusion, we developed and validated a reinforcement learning model to optimize the dose of dexmedetomidine for the prevention of delirium in ICU settings. Although our finding suggests that our model has the potential to support clinicians in sequential decision-making regarding drug dosing, the effective sample size was eight patients which indicates high uncertainty of our model’s validation. Therefore, further OPEs with larger samples are required to achieve a sufficient effective sample size and demonstrate the model’s benefits over clinicians’ policies before advancing to prospective studies.

## Methods

### Study design and databases

All data for model development were retrieved from the prospective registry of critically ill patients at the Seoul National University Hospital (SNUH) via clinical data warehouse (Supreme 2.0, Seoul, Republic of Korea), approved by its Institutional Review Board (IRB) (approval number: 2107-258-1246). The IRB also approved the retrospective analysis of this data (approval number: 2308-002-1453), with a waiver for written informed consent due to the study’s retrospective design and data anonymity.

For external validation, the Salzburg Intensive Care database (SICdb), which contains over 27 thousand admissions from four different ICUs at the University Hospital Salzburg, was used^46^. The SICdb offers both aggregated once-per-hour and highly granular once-per-minute data, including deidentified patient demographics, vital signs, laboratory tests, and medication information. The approval was obtained for 3rd party re-use of SICdb data for research from its steering group, and the research was conducted according to the data use agreement.

### Patient cohorts

Data from all patients admitted in medical or surgical ICUs between January 2008 and March 2023 from the derivation cohort (SNUH) were collected for model development and internal validation. Patients from the external validation cohort between 2013 and 2021 were included. Among both cohorts, those who received dexmedetomidine and had a target RASS between –2 and 0 were eligible, as our reinforcement learning model was designed to maintain light sedation within this range. Because dexmedetomidine is not appropriate for patients requiring deep sedation^47^, those who initially required prolonged deep sedation, defined as RASS values of −4 or −5, or propofol ≥30 mcg/kg/min for more than 24 consecutive hours, were not considered eligible.

### Exclusion criteria

Patients with the following characteristics were excluded:

In both cohorts:

- Age <18 years old at the time of ICU admission
- Length of ICU stay <1 or > 30 days
- Use of extracorporeal membrane oxygenation

In the derivation cohort:

- Diagnosis of delirium after hospitalization but before ICU admission.

In the external validation cohort, hospitalization times were unavailable, thus precluding the exclusion of patients diagnosed with delirium post-hospitalization but prior to ICU admission.

### Data extraction and preprocessing

In the derivation cohort, we obtained 49 items related to demographics, vital signs, ventilator-related variables, laboratory tests, pain severity scores, GCS and RASS scores, Confusion Assessment Method in the Intensive Care Unit (CAM-ICU), medication administration records, procedure records, clinical progress notes, and medical consultation notes. For the external validation cohort, 40 items were obtained; however, pain severity scores, sedation and consciousness assessments, clinical notes, and certain laboratory data were not available. A comprehensive list of the collected items is provided in Supplementary Table 1.

The GCS scores for eye opening, verbal response, and motor response were summed and used as a single score. In cases where one of the three indicators was missing, a conversion table was used to estimate and sum the scores^48^. The presence of pain was determined if either the numeric pain rating scale or the critical care non-verbal pain scale were greater than zero. Dexmedetomidine was collected as a numerical value, and all other medication information was collected in binary form. The dexmedetomidine doses were segmented into 15 uniform intervals, each increasing by 0.1 mcg/kg/h, spanning from 0 to 1.5 mcg/kg/h. This segmentation aligns with the standard clinical increments for dexmedetomidine administration, where doses are typically adjusted by 0.1 mcg/kg/h^16^. Remifentanil and sufentanil were combined into a single binary representation because of their similar effects^49,50^. For the numerical features, outliers were removed based on the upper and lower limits of physiological plausibility, as described in a previous study^51^, and listed in Supplementary Table 2.

Each admission data was represented as a multidimensional discrete time series with 6-h timesteps. When multiple measurements were present within a 6-h timestep, the median value was calculated. For timesteps lacking data, we initially imputed missing values using time-weighted average interpolation to leverage the temporal dynamics of our datasets. However, this method was not applicable when timesteps at the beginning or end of admissions lacked adjacent data points. In these instances, the remaining missing values were imputed using multivariate imputation, which utilizes available data from other variables. The rates of missingness for each variable among 6-h timesteps, and the mean and median measurement intervals for each variable are presented in Supplementary Tables 3 and 4, respectively. The follow-up period for each patient trajectory was defined as the time from initial dexmedetomidine administration to the time of ICU discharge or the time of starting prolonged deep sedation.

### Definition of delirium

The definition of delirium includes any of the following criteria being satisfied^52^: (1) positive CAM-ICU findings, (2) diagnosis made by physicians from the department of psychiatry, (3) administration of antipsychotics to treat delirium, and (4) clinical suspicion by the attending physician. CAM-ICU findings were obtained from the clinical data warehouse of SNUH. CAM-ICU was performed by trained bedside registered nurses once per 8 h nurse shift and has been shown to have reasonable inter-rater reliability, sensitivity, and specificity^53^. The second, third, and fourth criteria were identified from the medical consultant notes, medication administration records, and clinical progress notes, respectively. Two intensivists independently conducted the reviews. However, because only medication administration records could be obtained from the SICdb, only the third criterion was applied. For the third criterion, antipsychotics for delirium primarily included quetiapine and haloperidol in both datasets. Owing to the different use of antipsychotics between the two datasets that clonidine is also commonly used for delirium in European countries, and it was added to the third criterion^54,55^. After identifying all occurrences of delirium, we defined the onset of delirium as the initial occurrence of delirium during the ICU stay. Patients diagnosed with delirium after hospitalization but before ICU admission, were not considered to have delirium onset.

### Feature importance

The feature importance was determined using the SHAP method, which is based on game theory and provides importance scores for each feature^56^. Shapley values indicate a quantitative association between a feature and a given model output, with high Shapley values indicating an association with a high model output, and vice versa. This method has been used in medical research to visualize complex relationships captured by machine learning. Specifically, we utilized the LightGBM^57^, a gradient boosting framework that uses tree-based learning algorithms, to develop two separate prediction models: one predicting the clinicians’ actions and another for the AID actions based on state features. Each model was trained with the respective actions as the target variable to assess how various state features influenced the decision-making process. Subsequently, SHAP plots were generated from these trained models to visualize the feature importance. Therefore, this method was used in our study to determine how each variable in the state space contributed to our policy.

We also performed a subgroup SHAP analysis, stratified by the matching between AID and clinicians’ policy and the occurrence of delirium, resulting in four distinct subgroups: (1) policy-matched cases without delirium, (2) policy-unmatched cases with delirium, (3) policy-unmatched cases without delirium, and (4) policy-matched cases with delirium. For each subgroup, we trained separate LightGBM models and generated SHAP plots to compare feature importance across different scenarios.

To understand the combination of feature importance in dosage decision-making, we employed a PCA-based SHAP analysis approach. Specifically, we first conducted PCA on the state features and extracted the principal component sets that explain 90% of the cumulative variance of the data. We then performed SHAP analysis on these principal components. Finally, we converted back the important principal components ranked by their mean absolute SHAP values, and examined the feature loadings of these principal components to identify the contributions of feature combinations.

### Building the computational model

This study used a reinforcement learning algorithm after formulating the problem of treatment decision-making on the patient trajectory as a Markov decision process (MDP). The MDP comprises states, actions, rewards, and discount factors. The state and action are a set of all possible patient conditions and the finite set of possible actions that can be taken from a given state, and in our study, it represents the administered dose of dexmedetomidine.

The reward was formulated based on the primary aim of our model, which was to determine the optimal policy for preventing delirium. Specifically, we assign a penalty of −1 if delirium occurs during any given time points, a reward of 1 at the terminal state if no delirium occurred throughout the ICU stay, and 0 for all other time points. We designed our model to maximize the cumulative reward. The discount factor (*γ*) defines how much importance is given to future rewards compared to the reward in the current state. We set *γ* to 0.99, emphasizing the importance of future rewards to ensure consistent management of delirium risks both immediately and long after the initial administration of dexmedetomidine.

The derivation cohort was divided into 5 folds for cross-validation. In each fold, the data were split by patient-level into training (70%), validation (10%), and test (20%) sets. Within each of the 5 cross-validation loops, the individual test set (that is, the spatially separated partition) remained untouched throughout model development and the validation set was used to validate the fitting progress and checkpoint selection^58,59^. The checkpoint yielding the highest 95% lower bound of estimated performance return on the validation set was selected as the final checkpoint for each model. Subsequently, we obtained the selected models’ suggested actions on the individual test sets and aggregated them for downstream analysis. Finally, we trained our model on the entire derivation cohort and applied the final model to the external validation cohort.

### Estimation of the AID policy

Among reinforcement learning algorithms, offline reinforcement learning methods using a fixed dataset of trajectories with no environmental interactions have been used in the medical field. This method can optimize policies using retrospectively collected datasets, including clinicians’ decision-making regarding the dynamic conditions of patients in real-world settings. CQL^60^, an offline reinforcement learning method that learns a value function to estimate the performance of a policy while addressing the distributional shift between the dataset and the learned policy. CQL differs from standard Q-learning by mitigating the potential overestimation of unseen actions that can occur in offline settings due to the lack of interaction between the learned policy and the environment. CQL adds a regularizer to the loss function that explicitly minimizes the expected Q-values over actions that lie outside the training distribution, thereby reducing over-optimistic value estimations and improving the stability and reliability of policy evaluation in offline settings. This makes CQL more suitable for offline reinforcement learning in clinical settings, yielding good performance in solving some clinical problems such as mechanical ventilation control and drug dosing^61,62^. The training process for learning the optimal policy using CQL is described in the following sections.

Our model was trained using the CQL algorithm, optimizing a loss function that ensures that the state-action values under the current policy remain conservative, thereby preventing overestimation while integrating standard temporal difference learning from the Double Deep Q-Network^63^. The Double Deep Q-Network architecture uses two separate neural networks to decouple action selection from value estimation, promoting more stable and accurate learning of Q-values. The loss function we adopted is as follows:

1 $$L\left(\theta \right)={{\mathbb{E}}}_{{s}_{t} \sim D}\left[\log \mathop{\sum }\limits_{a}\exp Q\left({s}_{t},a\right)-{{\mathbb{E}}}_{a \sim D}\left[Q\left({s}_{t},a\right)\right]\right]+{L}_{{DoubleDQN}}(\theta )$$

2 $$\begin{array}{l}{L}_{{DoubleDQN}}\,\left(\theta \right)={{\mathbb{E}}}_{{s}_{t},{a}_{t},{r}_{t+1},{s}_{t+1} \sim D}\left[{\left({r}_{t+1}+\gamma {Q}_{{\theta }^{{\prime} }}\left({s}_{t+1},{argma}{x}_{a}{Q}_{\theta }\left({s}_{t+1},a\right)\right)-{Q}_{\theta }\left({s}_{t},{a}_{t}\right)\right)}^{2}\right]\end{array}$$

This loss function (Eq. 1) encapsulates the log-sum-exp of Q-values for regularization, maintaining adherence to the behavior policy’s distribution; the expected Q-value, ensuring that actions from the dataset are realistically valued; and the Double Deep Q-Network loss (Eq. 2), which assists in learning a stable and accurate Q-value function by utilizing two separate neural networks to decouple action selection from value estimation. In Eq. 2, when ${s}_{t}$ is the terminate state, the Q-value ${Q}_{\theta }\left({s}_{t},{a}_{t}\right)$ is updated using only the intermediate reward ${r}_{t+1}$, since there is no next state to estimate a value for.

To estimate the state-action values, a three-layer multilayer perceptron with 256 hidden dimensions was used. The model was trained using backpropagation of errors with a batch size of 4096 for 2000 epochs using the Adam optimizer at a learning rate of 6.25e-05. All the learning processes were conducted on an NVIDIA V100 GPU.

### Evaluation of AID and clinicians’ policy

To provide a comprehensive and unbiased assessment of the AID policy’s performance, we employed both FQE and WIS methods for OPE. FQE^64^ is a model-based approach that estimates the Q-function of the target policy using historical data^65^. WIS^66^ is a model-free OPE technique that estimates the value of a policy by weighting the importance of each sample based on the ratio of the evaluated policy to the behavior policy. For the WIS estimates, we developed multinomial logistic regression models to approximate the clinicians’ policy and softened the AID policy by assigning a high probability (0.99) to the recommended action and distributing a total probability of 0.01 among the remaining actions^67^. To enhance the robustness of our WIS estimates within the causal framework^68,69^, we incorporated potential confounders into the propensity model based on previous clinical studies, including age^70–73^, sex^74–77^, body mass index^78–80^, continuous renal replacement therapy^70,81–84^, mechanical ventilation^10,85,86^, and shock^38,87–90^. We also report the effective sample size, as it can significantly impact the reliability of the model evaluation^17^. To estimate the effective sample size, we used the methods proposed in previous studies^91,92^.

To estimate the CI of the performance return for each policy, we employed a bootstrapping method with the FQE and WIS algorithms^93,94^. For a conservative comparison, we compared the 95% lower bound of the AID performance return with the 95% upper bound of the clinicians’ returns, as in previous studies^18,45^. Additionally, we estimated the FQE and WIS values of a random policy for comparison.

### Statistical analysis

Python 3.8.0 (Python Software Foundation, Wilmington, DE, USA) was used for signal preprocessing, model development and validation, statistical testing, and visualization. The Python library “d3rlpy” was used for reinforcement learning model development and validation. For the comparison of dexmedetomidine doses between different patient groups under both the clinicians’ and AID policies, the Mann–Whitney U test was utilized. This test specifically assessed whether the distributions of doses for patients who developed delirium differed significantly from those who did not, under each policy. To categorize cases as ‘policy-matched’ or ‘policy-unmatched’ for subgroup analysis, we calculated the per-case mean of absolute dosing differences across all timepoints between clinicians’ and AID policies. We then classified cases based on whether their mean values fell below or above the overall first quantile of these differences. For the statistical analysis of patient characteristics, categorical variables were analyzed by proportional differences using the chi-square test or Fisher’s exact test. The *t*-test and Wilcoxon rank-sum test were used to compare the continuous and ordinal variables, respectively. Pearson correlation coefficients were calculated to identify the associations among state variables. All statistics for continuous variables are reported as either point estimates and 95% CIs or interquartile ranges. By contrast, statistics for categorical variables are reported as counts (frequencies) or proportions. A *P* < 0.05 was considered statistically significant.

## Supplementary information

SUPPLEMENTAL MATERIAL

## Data Availability

The data supporting this study’s findings are available from the corresponding author upon reasonable request.

## Code availability

The code used in this study can be accessed at https://github.com/SoominChung/AID.
