# Supplementary material for: Reinforcement learning model for optimizing dexmedetomidine dosing to prevent delirium in critically ill patients
Source: NPJ Digit Med. 2024 Nov 18;7:325. doi: 10.1038/s41746-024-01335-x (PMC11574043; doi:10.1038/s41746-024-01335-x)
Supplement: Supplementary file 1 — SUPPLEMENTAL MATERIAL [file 41746_2024_1335_MOESM1_ESM.pdf]

Supplementary Table 1. Collected items in the derivation and external validation cohorts

Category	Items	Derivation cohort	External validation cohort
Demographics	Age	O	O
Demographics	Gender	O	O
Demographics	Weight	O	O
Demographics	Height	O	O
Laboratory	HCO_3	O	O
Laboratory	pCO_2	O	O
Laboratory	pH	O	O
Laboratory	pO2	O	O
Laboratory	PT	O	O
Laboratory	Sodium	O	O
Laboratory	Creatinine	O	O
Laboratory	Potassium	O	O
Laboratory	BUN	O	O
Laboratory	Hb	O	O
Laboratory	WBC	O	O
Laboratory	PLT	O	X
Laboratory	hsCRP	O	O
Laboratory	glucose	O	O
Laboratory	bilirubin	O	O
Ventilator-related variable	F_IO2	O	O
Glasgow coma scale	GCS	O	X
Vital	BT	O	O
Vital	ADBP	O	O
Vital	DBP	O	O
Vital	Heart rate	O	O
Vital	RR	O	O

Vital	ASBP	O	O
Vital	SBP	O	O
Vital	SpO_2	O	O
Drug	Remifentanil	O	O
Drug	Propofol	O	O
Drug	Norepinephrine	O	O
Drug	Dopamine	O	O
Drug	Dexmedetomidine	O	O
Drug	Vasopressin	O	O
Drug	Milrinone	O	O
Drug	Midazolam	O	O
Drug	Epinephrine	O	O
Drug	Morphine	O	O
Drug	Fentanyl	O	O
Pain evaluation	CNPS	O	X
Pain evaluation	NRS	O	X
Richmond Agitation-Sedation Scale	RASS	O	X
Confusion Assessment Method in the Intensive Care Unit	CAM-ICU	O	X
Procedure	Mechanical ventilation	O	O
Procedure	CRRT	O	O
Clinical progress note	Nursing notes	O	X
Clinical progress note	Clinical observation notes	O	X
Medical consultation notes	Medical consultation notes	O	X

HCO_3: bicarbonate; pCO_2: partial pressure of carbon dioxide; pO_2: partial pressure of oxygen; PT: prothrombin time; BUN: blood urea nitrogen; Hb: hemoglobin; WBC: white blood cell count; PLT: platelet count; hsCRP: high-sensitivity C-reactive protein; F_IO2: fraction of inspired oxygen; GCS:

Glasgow coma scale; BT: body temperature; ADBP: arterial diastolic blood pressure; DBP: diastolic blood pressure; RR: respiratory rate; ASBP: arterial systolic blood pressure; SBP: systolic blood pressure; SpO_2: oxygen saturation; CNPS: confusion numerical rating scale for pain; NRS: numeric rating scale; RASS: Richmond agitation-sedation scale; CAM-ICU: confusion assessment method for the intensive care unit; CRRT: continuous renal replacement therapy.

Supplementary Table 2. Upper and lower limits of physiological plausibility for numerical features

Variable	Lower limit	Upper limit
HCO_3	0.0	60.0
pCO_2	0.0	200.0
pH	6.3	8.4
pO2	32.0	700.0
PT	0.75	25.0
Sodium	50.0	225.0
Creatinine	0.1	60.0
Potassium	0.0	12.0
BUN	0.0	250.0
Hb	0.0	25.0
WBC	0.0	1000.0
PLT	0.0	2000.0
hsCRP	0.01	73.33
glucose	33.0	2000.0
bilirubin	0.1	60.0
F_IO2	21.0	100.0
BT	26.0	45.0
DBP	0.0	375.0
Heart rate	0.0	350.0
RR	0.0	300.0
SBP	0.0	375.0
SpO_2	0.0	100.0

RASS: Richmond agitation-sedation scale; HCO_3: bicarbonate; pCO_2: partial pressure of carbon dioxide; pO_2: partial pressure of oxygen; PT: prothrombin time; BUN: blood urea nitrogen; Hb: hemoglobin; WBC: white blood cell count; PLT: platelet count; hsCRP: high-sensitivity C-reactive protein; F_IO2: fraction of inspired oxygen; GCS: Glasgow coma scale; BT: body temperature; DBP: diastolic blood pressure; RR: respiratory rate; SBP: systolic blood pressure; SpO_2: oxygen saturation.

Supplementary Table 3. Proportion of missing values for time-varying variables measured among 6-hour timesteps

Variable	Missingness proportion (%)
HCO_3	11.475
pCO_2	11.475
pH	11.409
pO2	11.544
PT	98.613
Sodium	7.711
Creatinine	11.094
Potassium	7.711
BUN	11.115
Hb	9.807
WBC	11.313
PLT	14.006
hsCRP	19.019
bilirubin	17.073
glucose	36.304
GCS	2.386
F_IO2	33.824
DBP	1.453
BT	11.059
RR	8.592
Heart rate	1.718
SBP	1.453
SpO_2	1.527

HCO_3: bicarbonate; pCO_2: partial pressure of carbon dioxide; pO_2: partial pressure of oxygen; PT: prothrombin time; BUN: blood urea nitrogen; Hb: hemoglobin; WBC: white blood cell count; PLT: platelet count; hsCRP: high-sensitivity C-reactive protein; F_IO2: fraction of inspired oxygen; GCS: Glasgow coma scale; BT: body temperature; DBP: diastolic blood pressure; RR: respiratory rate; SBP: systolic blood pressure; SpO_2: oxygen saturation.

Supplementary Table 4. Mean and median measurement intervals for time-varying variables.

Category	Variable	Delta average (H:M)	Delta median (H:M)
Action	Dexmedetomidine	03:13	01:36
State	HCO_3	05:46	04:45
State	pCO_2	05:45	04:45
State	pH	05:44	04:43
State	pO2	05:45	04:45
State	PT	13:31	10:45
State	Sodium	06:27	05:03
State	Creatinine	11:07	07:50
State	Potassium	06:28	05:04
State	BUN	11:09	07:52
State	Hb	09:04	06:34
State	WBC	13:47	11:19
State	PLT	14:02	11:27
State	hsCRP	23:28	23:55
State	glucose	09:21	04:54
State	bilirubin	17:05	22:21
State	F_IO2	00:40	00:30
State	GCS	01:59	02:00
State	BT	00:59	01:00
State	DBP	00:41	01:00
State	Heart rate	00:41	01:00
State	RR	00:34	00:30
State	SBP	00:41	01:00
State	SpO_2	00:42	01:00
State	Remifentanil	03:11	02:00
State	Propofol	02:49	01:17
State	Norepinephrine	02:25	01:00
State	Dopamine	02:49	01:15

State	Vasopressin	03:23	01:54
State	Milrinone	03:00	03:00
State	Midazolam	04:05	02:00
State	Epinephrine	02:20	01:00
State	Morphine	02:44	02:00
State	Fentanyl	02:13	02:00
State	Presence of pain	03:10	04:00

RASS: Richmond agitation-sedation scale; HCO_3: bicarbonate; pCO_2: partial pressure of carbon dioxide; pO_2: partial pressure of oxygen; PT: prothrombin time; BUN: blood urea nitrogen; Hb: hemoglobin; WBC: white blood cell count; PLT: platelet count; hsCRP: high-sensitivity C-reactive protein; F_IO2: fraction of inspired oxygen; GCS: Glasgow coma scale; BT: body temperature; DBP: diastolic blood pressure; RR: respiratory rate; SBP: systolic blood pressure; SpO_2: oxygen saturation.

Supplementary Figure 1. Beeswarm plot to depict feature importance derived from the SHAP method. **a**. Feature importance of the AID policy. **b**. Feature importance of the clinicians' policy. F_IO2: fraction of inspired oxygen; BT: body temperature; GCS: Glasgow coma scale; RR: respiratory rate; DBP: diastolic blood pressure; hsCRP: high-sensitivity C-reactive protein; WBC: white blood cell; AID: Artificial Intelligence model for Delirium prevention; SHAP: SHapley Additive exPlanations.

a

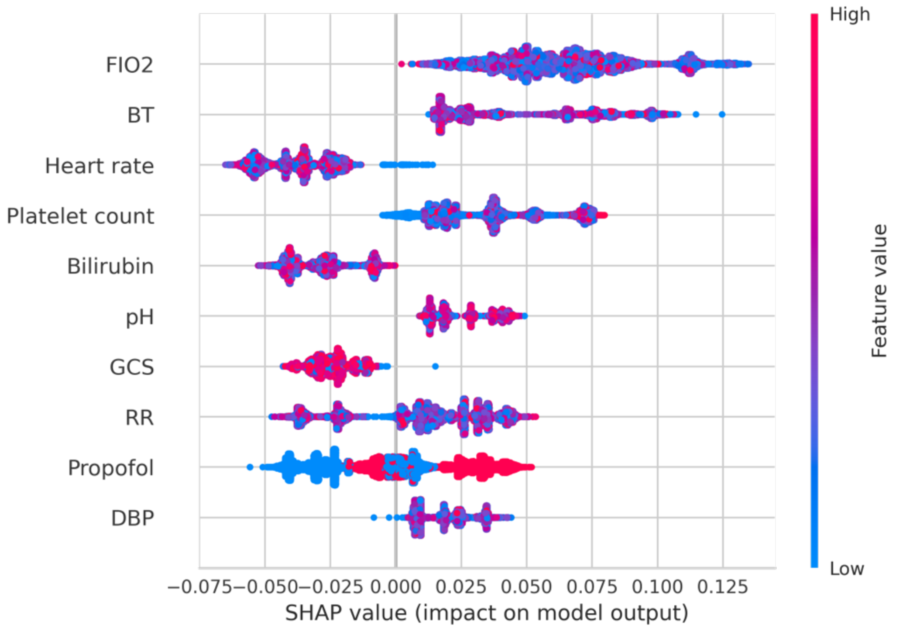

b

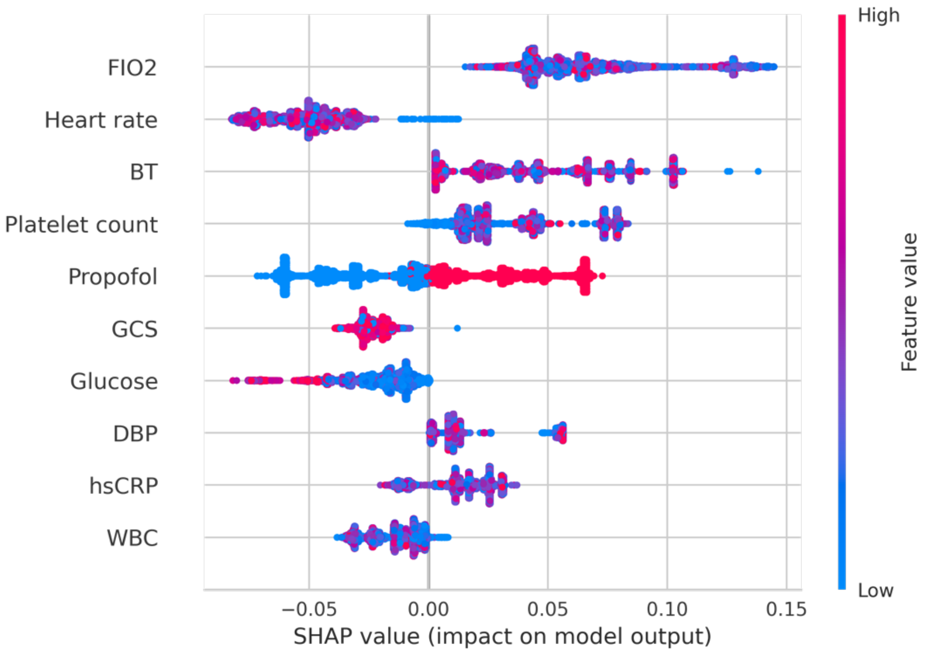

Supplementary Figure 2. Bar plot to depict feature importance derived from the SHAP method in the external validation cohort **a**. Feature importance of the AID policy. **b**. Feature importance of the clinicians' policy. F_IO2: fraction of inspired oxygen; hsCRP: high-sensitivity C-reactive protein; GCS: Glasgow coma scale; HCO_3: bicarbonate; BT: body temperature; DBP: diastolic blood pressure; BUN: blood urea nitrogen; WBC: white blood cell; AID: Artificial Intelligence model for Delirium prevention; SHAP: SHapley Additive exPlanations.

a

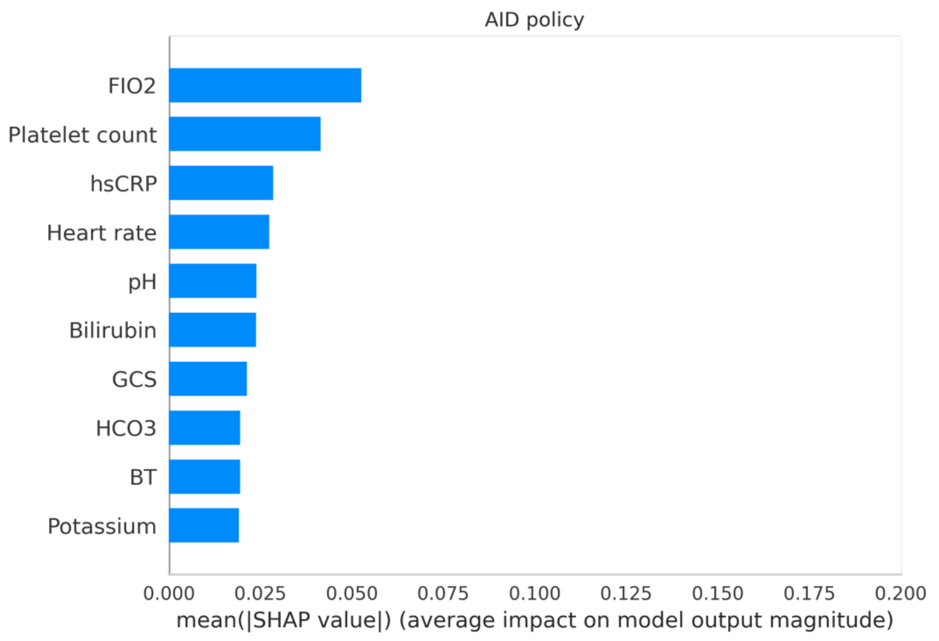

b

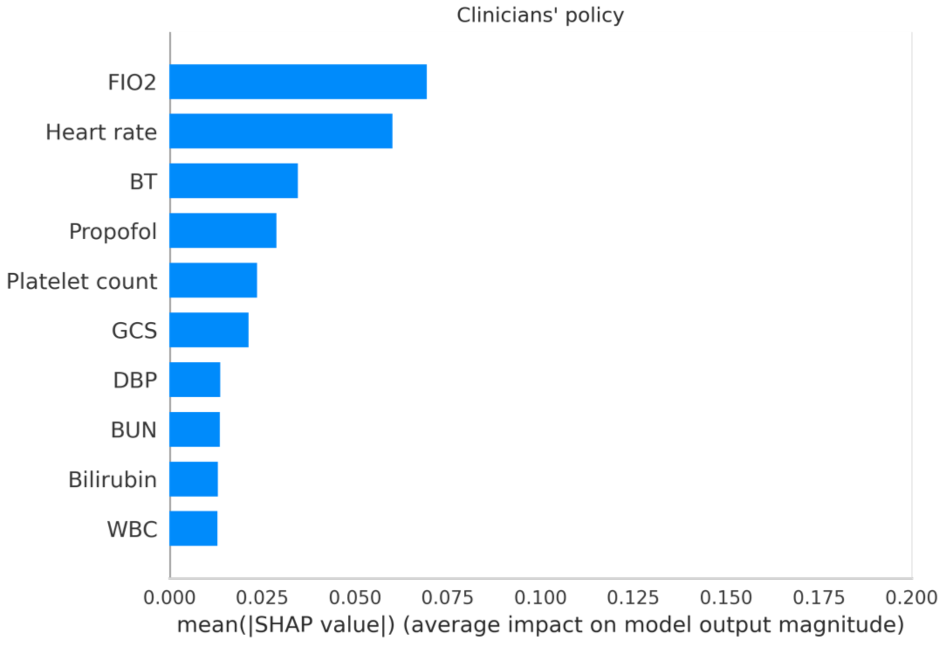

Supplementary Figure 3. Beeswarm plot to depict feature importance derived from the SHAP method in the external validation cohort **a**. Feature importance of the AID policy. **b**. Feature importance of the clinicians' policy. F_IO2: fraction of inspired oxygen; hsCRP: high-sensitivity C-reactive protein; BT: body temperature; GCS: Glasgow coma scale; RR: respiratory rate; DBP: diastolic blood pressure; WBC: white blood cell; AID: Artificial Intelligence model for Delirium prevention; SHAP: SHapley Additive exPlanations.

a

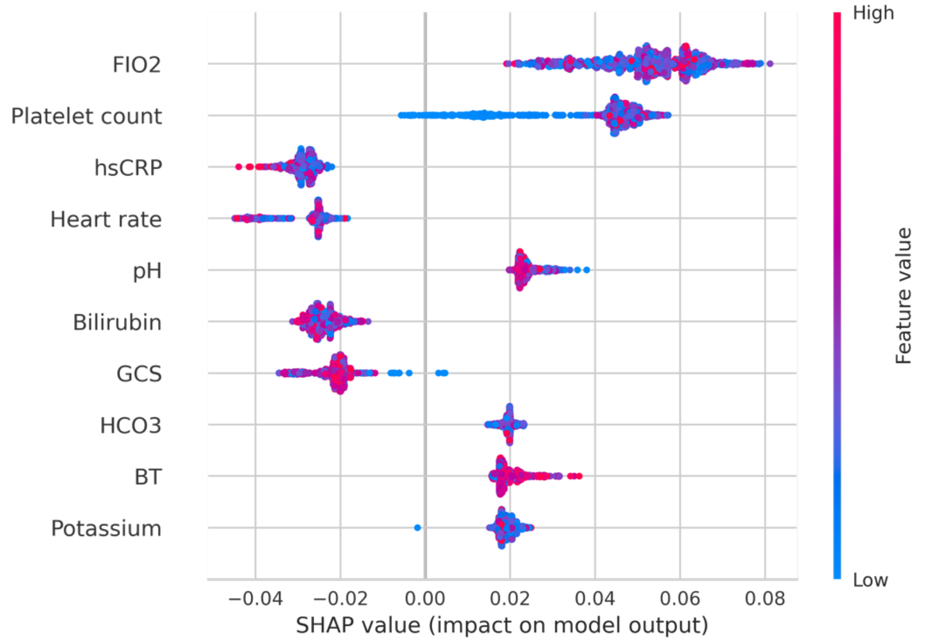

b

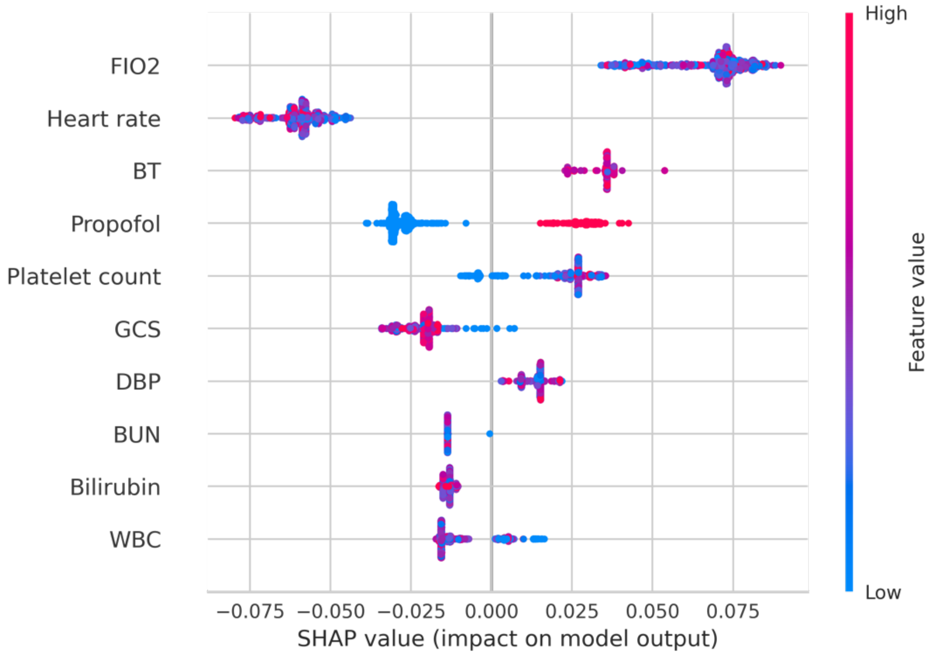

Supplementary Figure 4. Feature importance derived from the SHAP method for subgroups stratified by policy matching and delirium occurrence in the external validation cohort **a.** Policy-matched subgroup without delirium. **b.** Policy-unmatched subgroup with delirium. **c.** Policy-unmatched subgroup without delirium. **d.** Policy-matched subgroup with delirium. BT: body temperature; DBP: diastolic blood pressure; F_IO2: fraction of inspired oxygen; BUN: blood urea nitrogen; GCS: Glasgow coma scale; Hb: hemoglobin; BT: body temperature; RR: respiratory rate; WBC: white blood cell; PT: prothrombin time; HCO_3: bicarbonate; SBP: systolic blood pressure; HCO_3: bicarbonate; AID: Artificial Intelligence model for Delirium prevention; SHAP: SHapley Additive exPlanations.

a

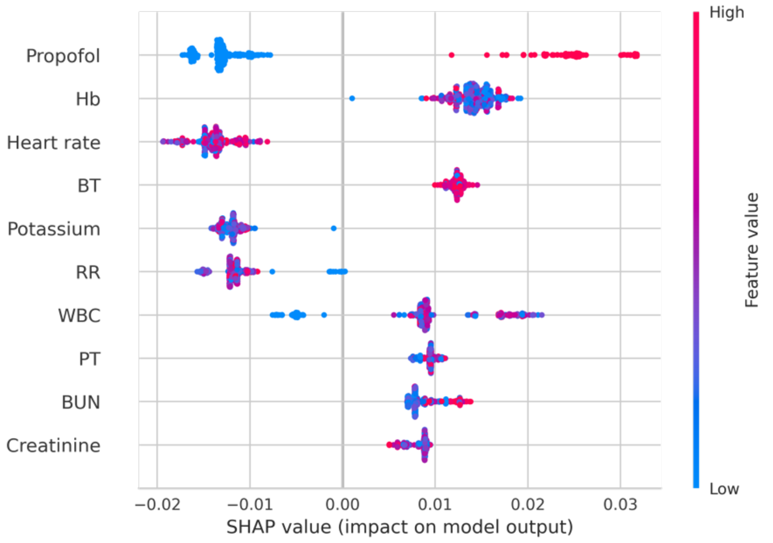

b

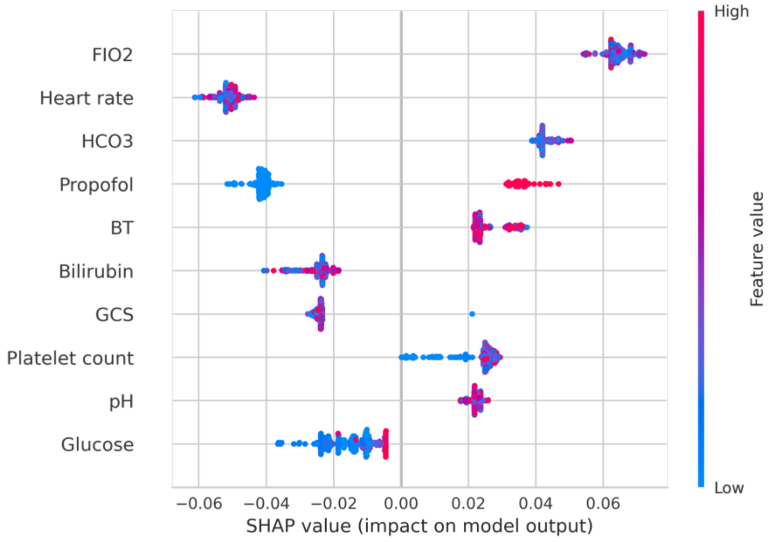

c

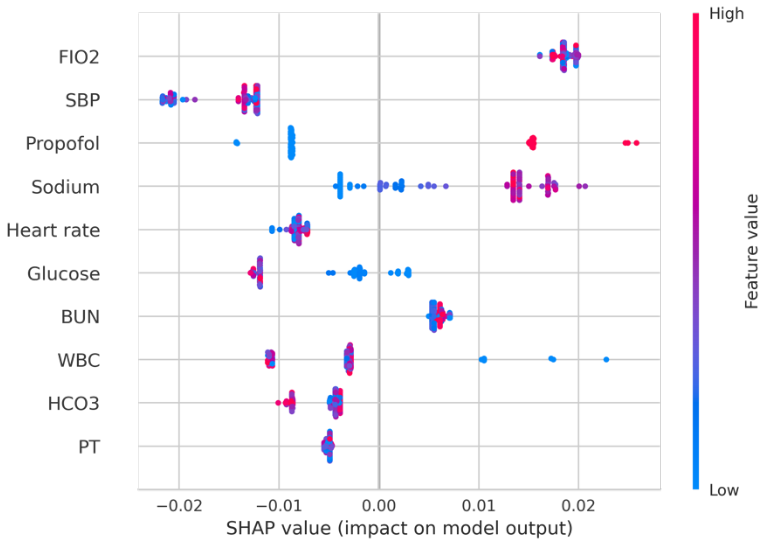
FIO2
SBP
Propofol
Sodium
Heart rate
Glucose
BUN
WBC
HCO3
PT
−0.02
−0.01
0.00
0.01
0.02
SHAP value (impact on model output)
High
Low
Feature value

d

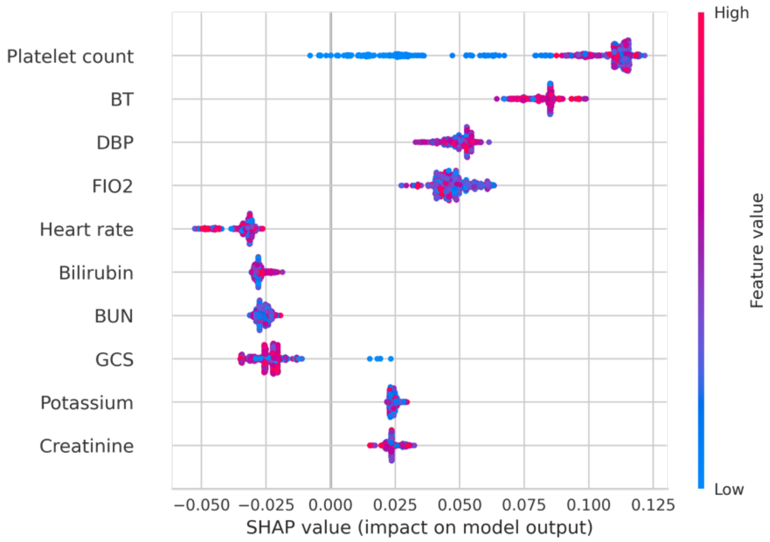
Platelet count
BT
DBP
FIO2
Heart rate
Bilirubin
BUN
GCS
Potassium
Creatinine
−0.050
−0.025
0.000
0.025
0.050
0.075
0.100
0.125
SHAP value (impact on model output)
High
Low
Feature value

Supplementary Figure 5. Pair plots among the top five features derived from the SHAP analysis. The derivation cohort was divided into four subgroups, stratified by policy matching and delirium occurrence. The hue represents the absolute error between clinicians' actions and AID actions. **a.** Policy-matched subgroup without delirium. **b.** Policy-unmatched subgroup with delirium. **c.** Policy-unmatched subgroup without delirium. **d.** Policy-matched subgroup with delirium. F_IO2: fraction of inspired oxygen; BT: body temperature; SHAP: SHapley Additive exPlanations; AID: Artificial Intelligence model for Delirium prevention.

a

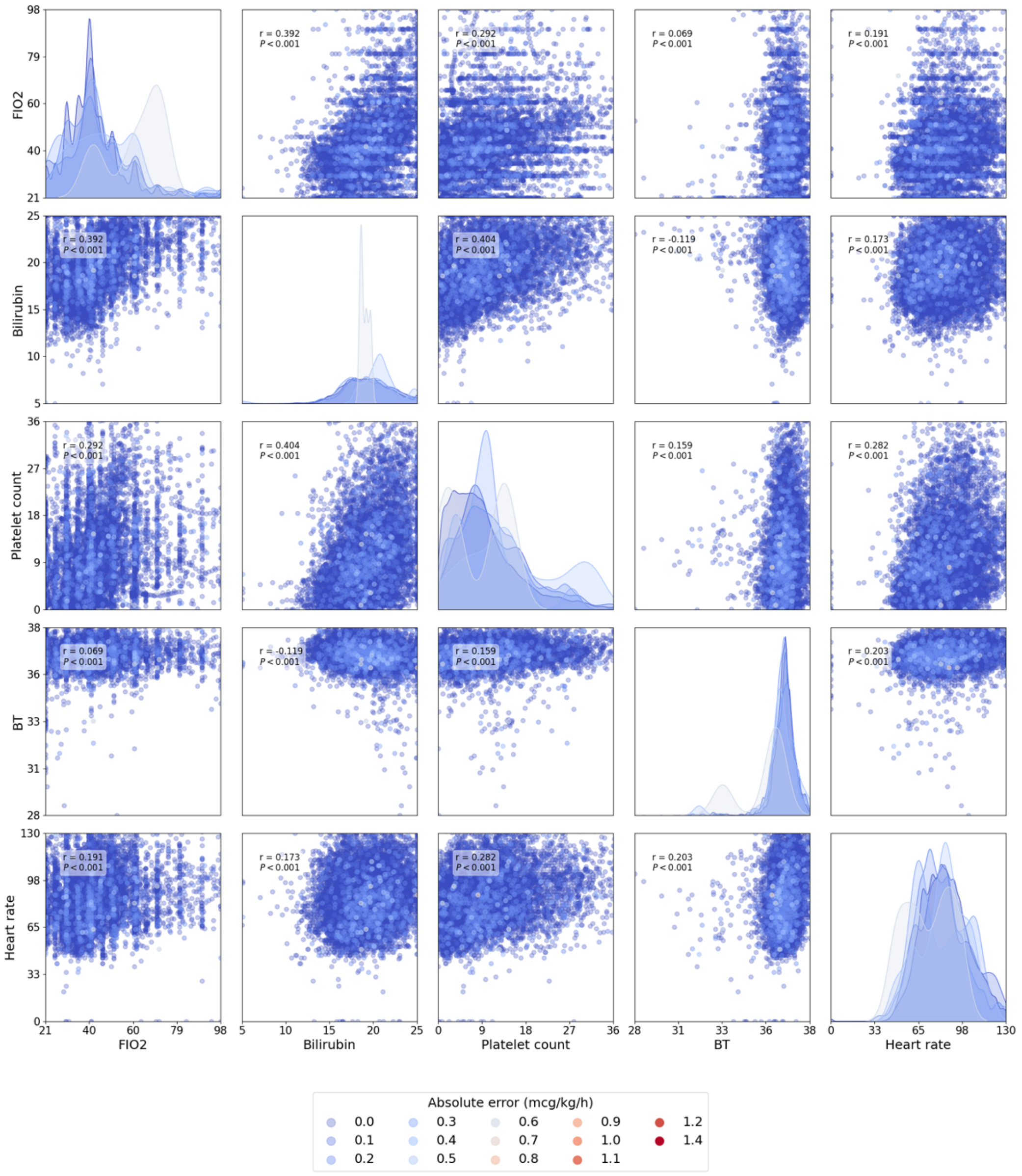

b

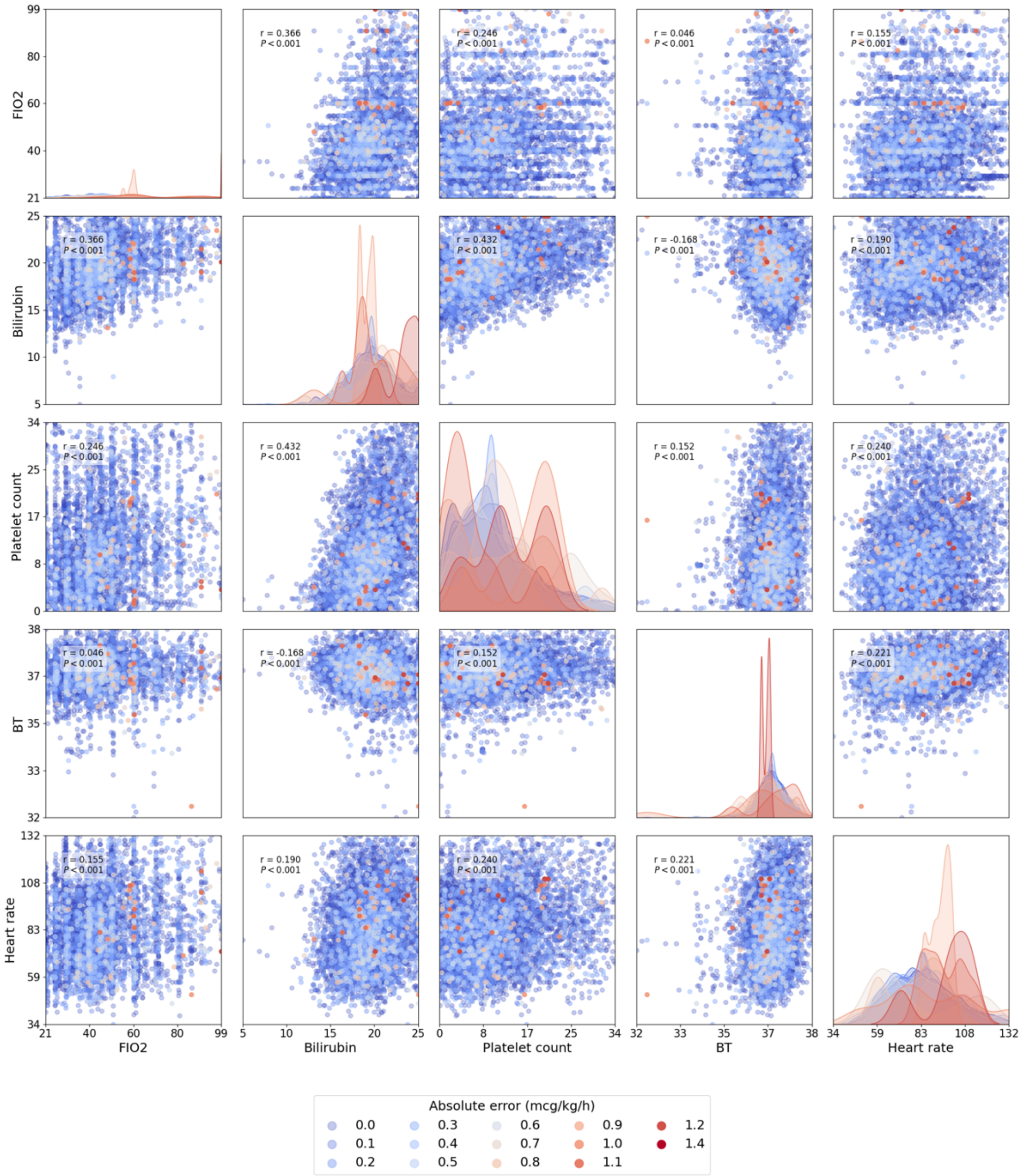

FIO2
Bilirubin
Platelet count
BT
Heart rate
r = 0.366
P < 0.001
r = 0.246
P < 0.001
r = 0.046
P < 0.001
r = 0.155
P < 0.001
r = 0.432
P < 0.001
r = -0.168
P < 0.001
r = 0.190
P < 0.001
r = 0.152
P < 0.001
r = 0.240
P < 0.001
r = 0.221
P < 0.001
Absolute error (mcg/kg/h)
0.0
0.1
0.2
0.3
0.4
0.5
0.6
0.7
0.8
0.9
1.0
1.1
1.2
1.4

c

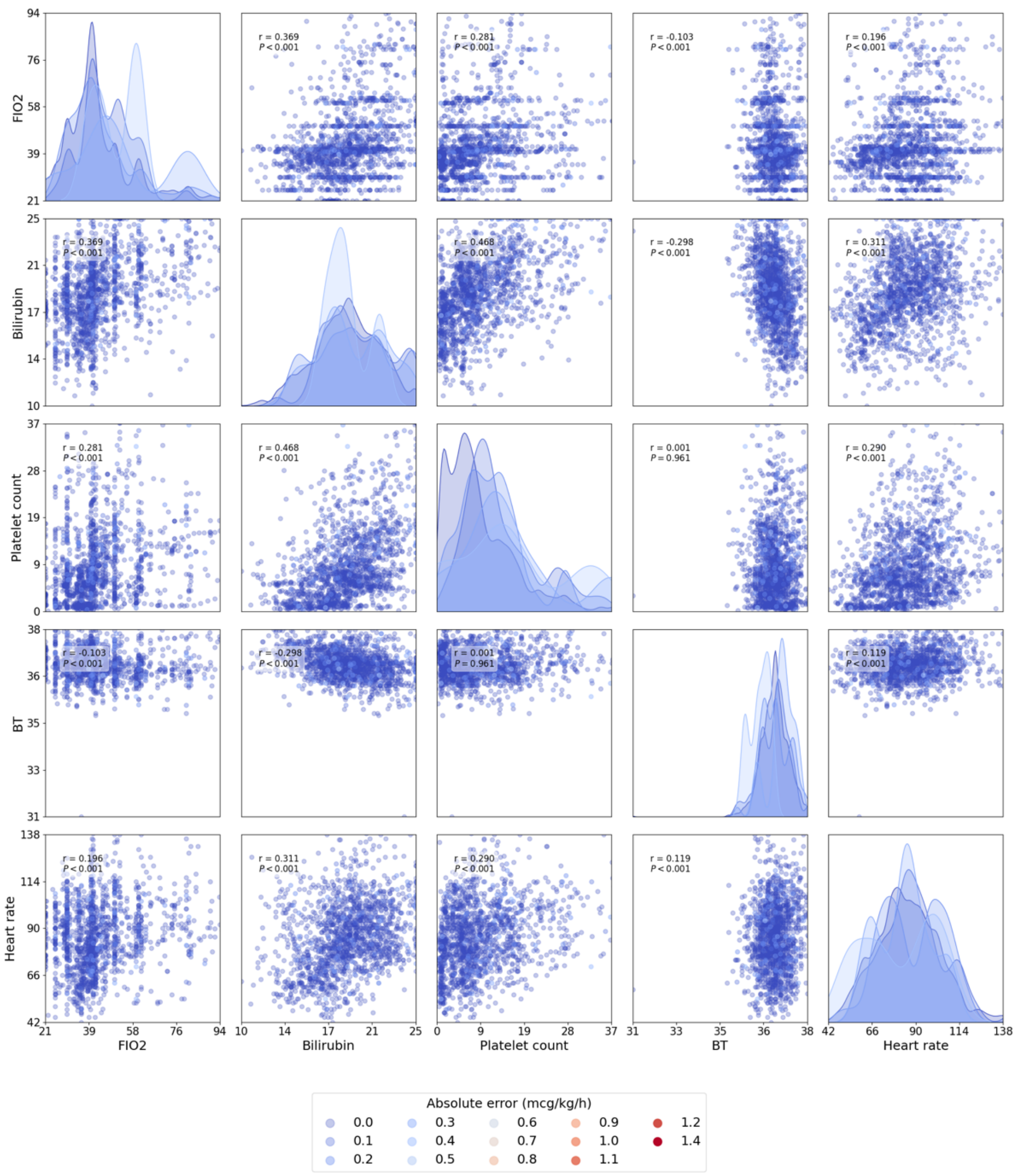
FIO2
Bilirubin
Platelet count
BT
Heart rate
94
76
58
39
21
25
21
17
14
10
37
28
19
9
0
38
36
35
33
31
138
114
90
66
42
21 39 58 76 94
10 14 17 21 25
0 9 19 28 37
31 33 35 36 38
42 66 90 114 138
r = 0.369
P < 0.001
r = 0.281
P < 0.001
r = -0.103
P < 0.001
r = 0.196
P < 0.001
r = 0.468
P < 0.001
r = -0.298
P < 0.001
r = 0.311
P < 0.001
r = 0.001
P = 0.961
r = 0.290
P < 0.001
r = 0.119
P < 0.001
Absolute error (mcg/kg/h)
0.0
0.1
0.2
0.3
0.4
0.5
0.6
0.7
0.8
0.9
1.0
1.1
1.2
1.4

d

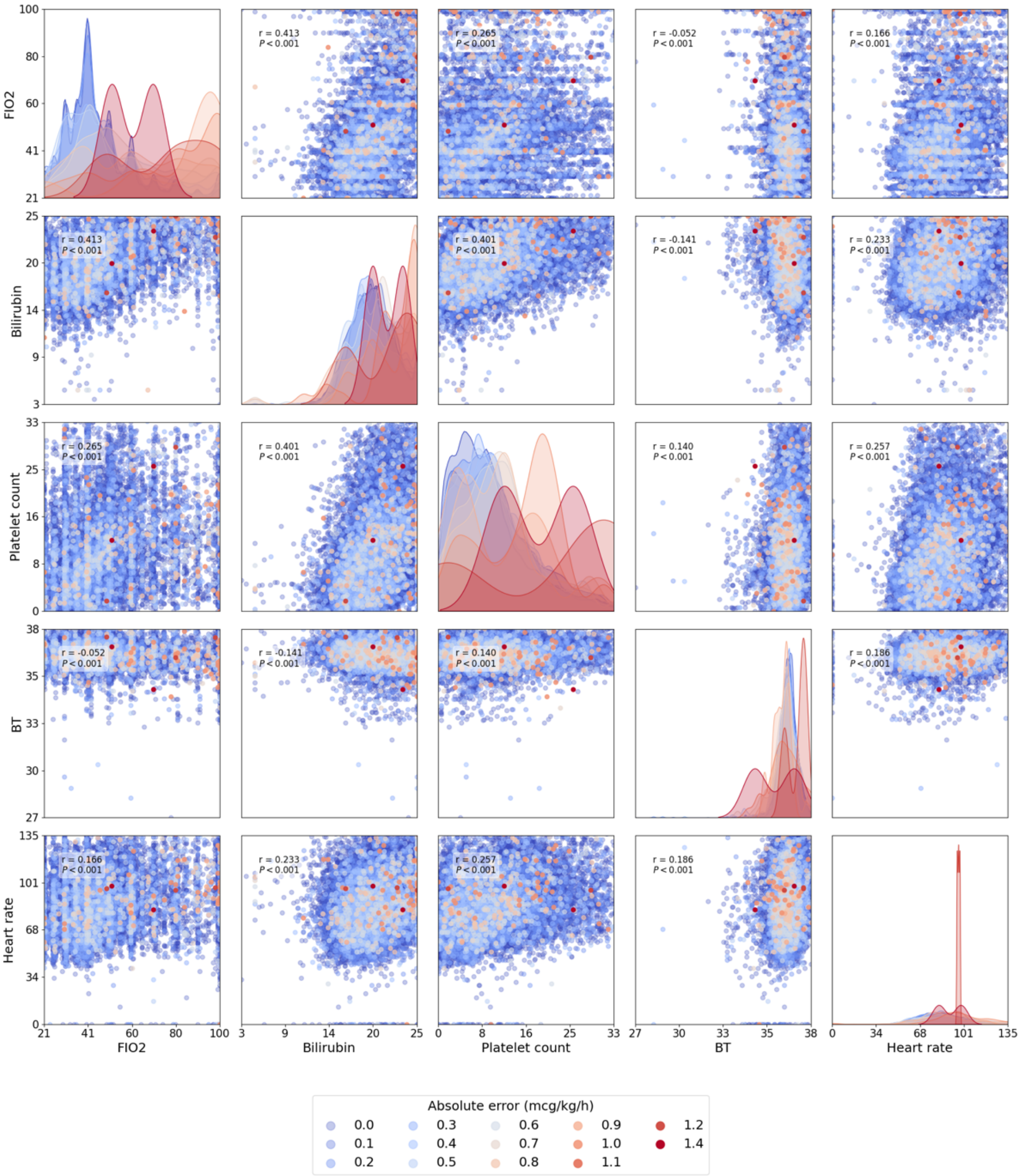
FIO2
Bilirubin
Platelet count
BT
Heart rate
Absolute error (mcg/kg/h)
0.0
0.1
0.2
0.3
0.4
0.5
0.6
0.7
0.8
0.9
1.0
1.1
1.2
1.4

Supplementary Figure 6. Pair plots among the top five features derived from the SHAP analysis. The external validation cohort was divided into four subgroups, stratified by policy matching and delirium occurrence. The hue represents the absolute error between clinicians' actions and AID actions. **a.** Policy-matched subgroup without delirium. **b.** Policy-unmatched subgroup with delirium. **c.** Policy-unmatched subgroup without delirium. **d.** Policy-matched subgroup with delirium. F_IO2: fraction of inspired oxygen; BT: body temperature; SHAP: SHapley Additive exPlanations; AID: Artificial Intelligence model for Delirium prevention.

a

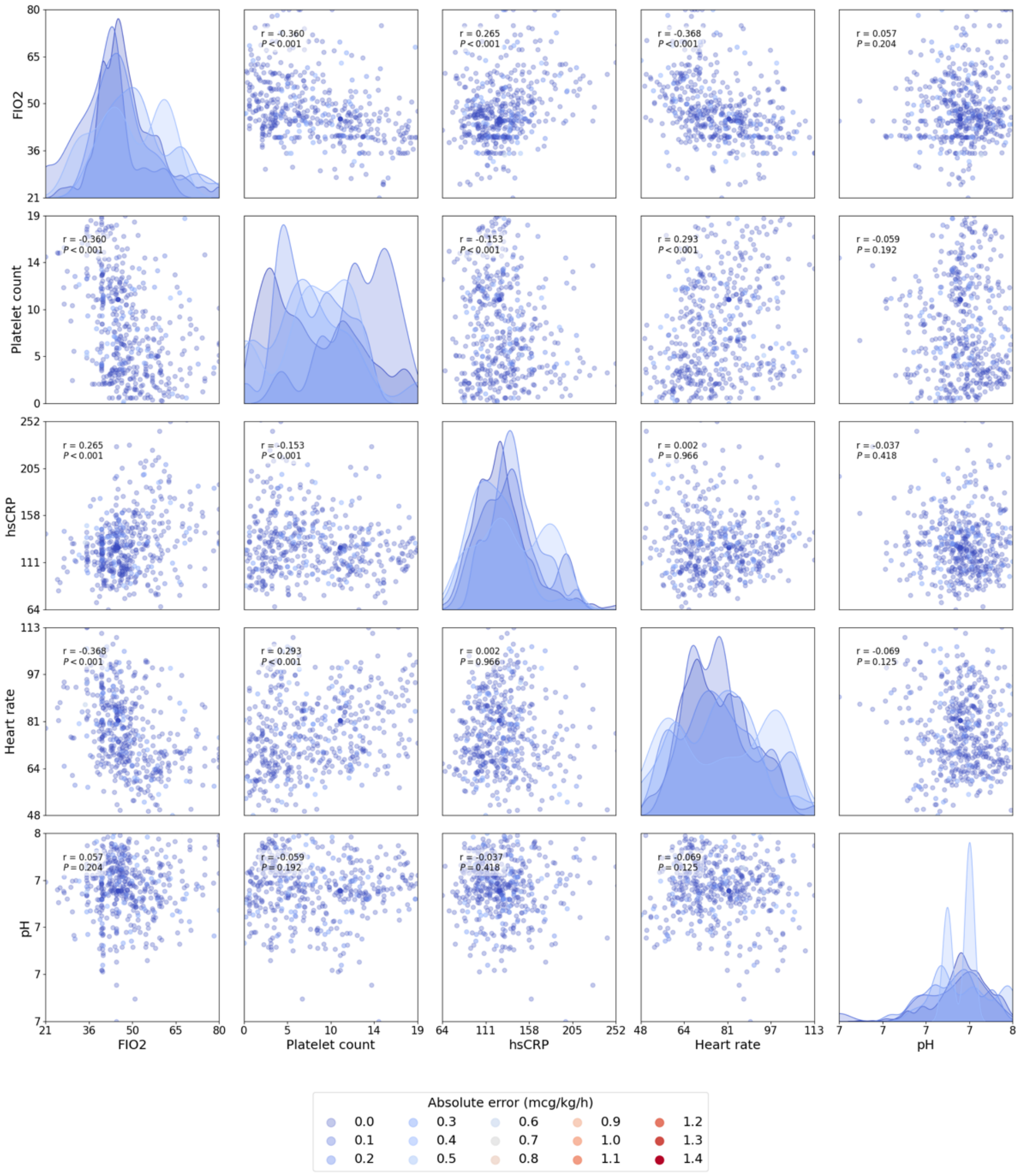

b

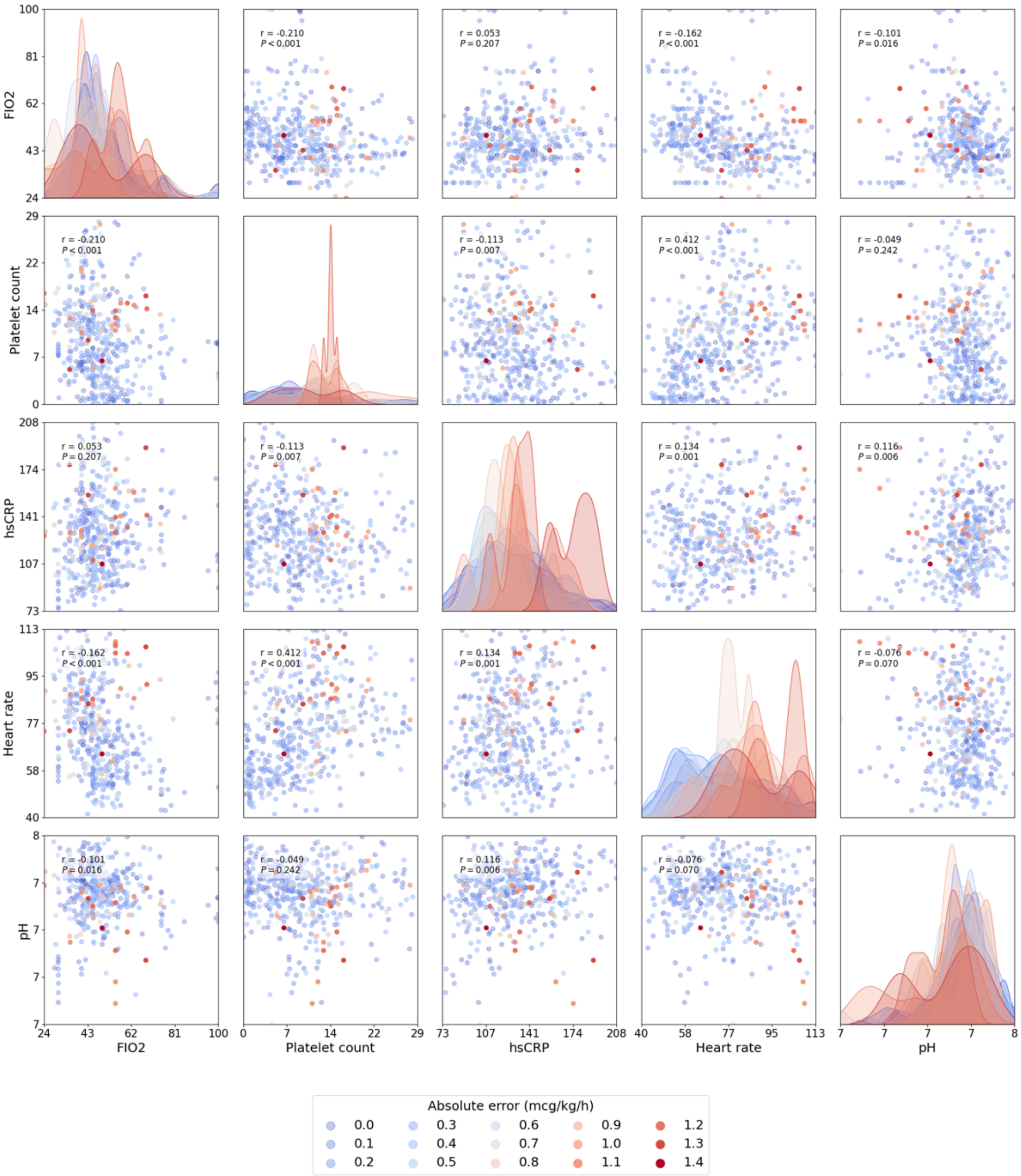
FIO2
Platelet count
hsCRP
Heart rate
pH
r = -0.210
P < 0.001
r = 0.053
P = 0.207
r = -0.162
P < 0.001
r = -0.101
P = 0.016
r = -0.113
P = 0.007
r = 0.412
P < 0.001
r = -0.049
P = 0.242
r = 0.134
P = 0.001
r = 0.116
P = 0.006
r = -0.076
P = 0.070
Absolute error (mcg/kg/h)
0.0
0.1
0.2
0.3
0.4
0.5
0.6
0.7
0.8
0.9
1.0
1.1
1.2
1.3
1.4

c

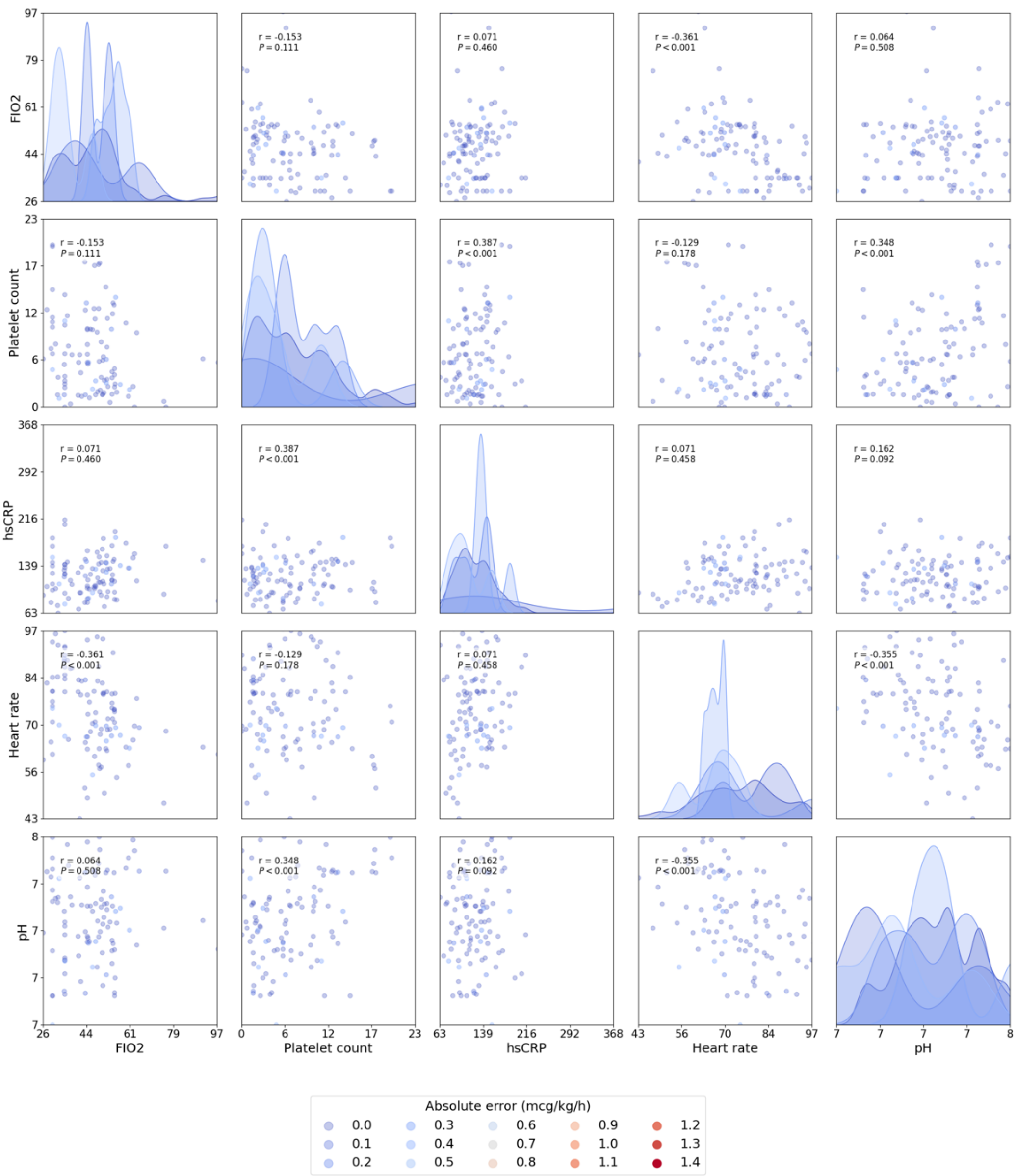

FIO2
Platelet count
hsCRP
Heart rate
pH
r = -0.153
P = 0.111
r = 0.071
P = 0.460
r = -0.361
P < 0.001
r = 0.064
P = 0.508
r = -0.153
P = 0.111
r = 0.387
P < 0.001
r = -0.129
P = 0.178
r = 0.348
P < 0.001
r = 0.071
P = 0.460
r = 0.387
P < 0.001
r = 0.071
P = 0.458
r = 0.162
P = 0.092
r = -0.361
P < 0.001
r = -0.129
P = 0.178
r = 0.071
P = 0.458
r = -0.355
P < 0.001
r = 0.064
P = 0.508
r = 0.348
P < 0.001
r = 0.162
P = 0.092
r = -0.355
P < 0.001
Absolute error (mcg/kg/h)
0.0
0.1
0.2
0.3
0.4
0.5
0.6
0.7
0.8
0.9
1.0
1.1
1.2
1.3
1.4

d

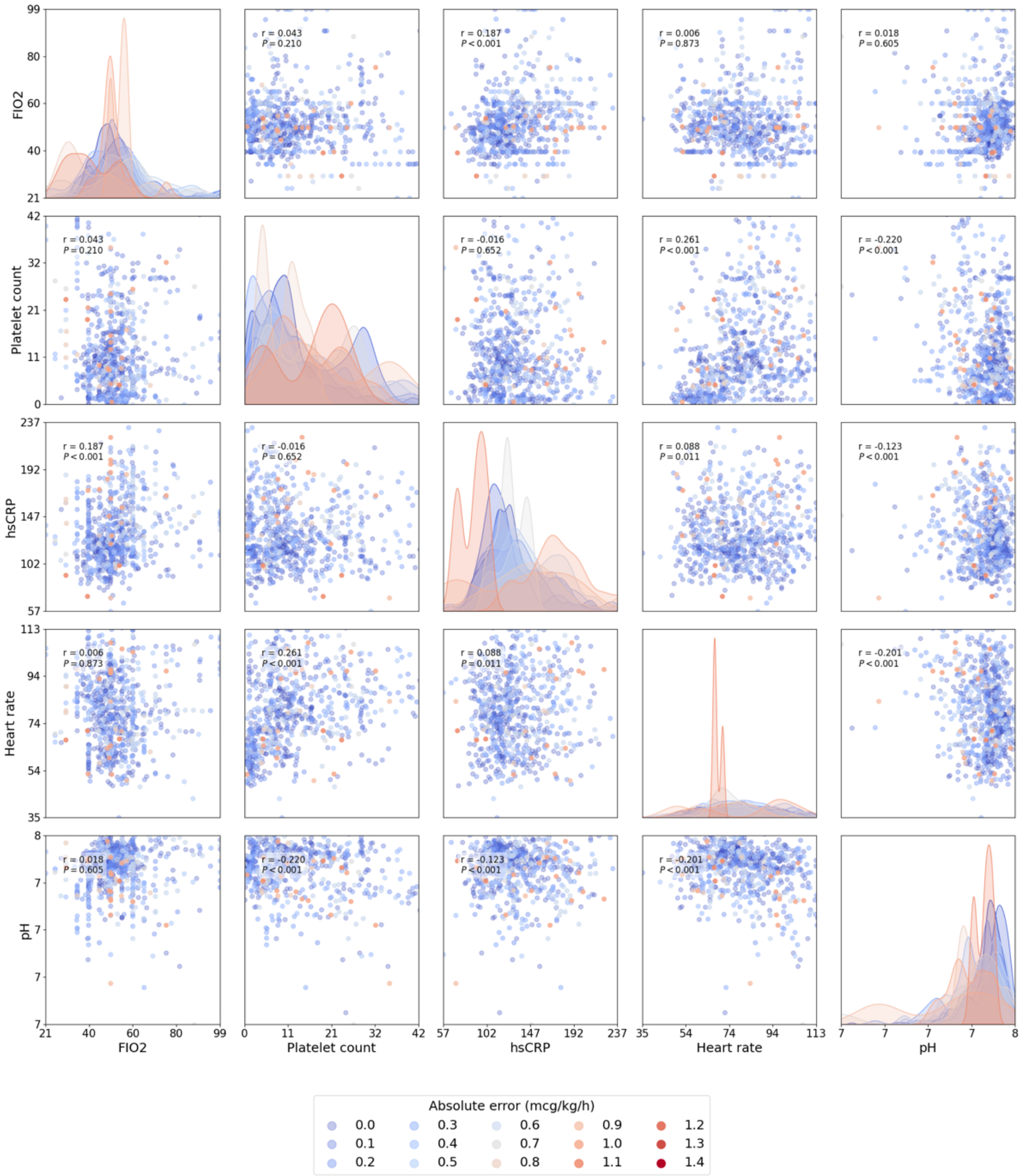
FIO2
Platelet count
hsCRP
Heart rate
pH
r = 0.043
P = 0.210
r = 0.187
P < 0.001
r = 0.006
P = 0.873
r = 0.018
P = 0.605
r = -0.016
P = 0.652
r = 0.261
P < 0.001
r = -0.220
P < 0.001
r = 0.088
P = 0.011
r = -0.123
P < 0.001
r = -0.201
P < 0.001
Absolute error (mcg/kg/h)
0.0
0.1
0.2
0.3
0.4
0.5
0.6
0.7
0.8
0.9
1.0
1.1
1.2
1.3
1.4
